# Oct4 cooperates with c-Myc to improve mesenchymal-to-endothelial transition and myocardial repair of cardiac-resident mesenchymal stem cells

**DOI:** 10.1186/s13287-022-03120-7

**Published:** 2022-09-02

**Authors:** Lan Zhao, Jianshuo Wang, Pengzhen Wang, Zhanyu Deng, Jin Cui, Weiguang Huang, Shaoheng Zhang

**Affiliations:** 1 Department Of Cardiology, GuangZhou Red Cross Hospital Medical College of Ji-Nan University, 396 Tongfuzhong Road, Haizhu District, Guangzhou, 510220 China; 2 Department of Cardiology, Dahua Hospital, 901 Laohumin Road, Xuhui District, Shanghai, 200237 China; 3 Guangzhou Institute of Traumatic Surgery, Guangzhou Red Cross Hospital, Jinan University, Guangzhou, Guangdong 510220 People’s Republic of China

**Keywords:** Myocardial Infarct, Angiogenesis, Mesenchymal stem cells, c-Myc, Oct4

## Abstract

**Background:**

Cardiac-resident mesenchymal stem cells (cMSCs) can exhibit fibrotic, proinflammatory, and proangiogenic phenotype in response to myocardial ischemia (Isch). How their phenotypic fate decisions are determined remains poorly understood. Here, we demonstrate that the cooperation of Oct4 and c-Myc in cMSCs creates a preferable mesenchymal-to-endothelial transition (MEndoT) to promote angiogenesis and consequent myocardial repair.

**Methods:**

We collected MSCs from cardiac and peripheral blood of rat with left ventricular Isch (LV Isch) 30 days after myocardial infarction (MI) or sham operation. After a comparison of characterization between cMSCs and peripheral blood MSCs (pbMSCs), we conducted transcriptome analysis and RNA sequencing of cMSCs. Using loss/gain-of-function approaches to understand the cooperation of c-Myc and Oct4 on MEndoT of cMSCs under hypoxic condition, we explored the mechanisms through transcriptome and functional experiment, and chromatin immunoprecipitation. Next, we transplanted male cMSCs with overexpression or inhibition of c-Myc/Oct4 into the infarcted myocardium of female rats and evaluated infarct size, cell retention, inflammation, remodeling, and function after 30 days.

**Results:**

LV Isch switched cMSCs toward both inflammatory and proangiogenic phenotypes, with increased secretion of inflammatory cytokines as well as decreased expression of proangiogenic factors. The effect of LV Isch on pbMSCs was less remarkable. Gene expression heatmap showed imbalance in expression of Oct4 and c-Myc regulating genes associated with remodeling of cMSCs. We provided evidence that cMSCs-specific c-Myc- versus Oct4-overexpression showed divergent genomic signatures, and their corresponding target genes play an important role in regulating cMSCs phenotypic changes. In particular, Oct4 accelerated angiogenesis induced by c-Myc overexpression in cMSCs and inhibited their phenotypic transition into inflammatory cells and fibroblast. Mechanistically, exogenous Oct4 caused c-Myc to translocate from the nucleus to the cytoplasm and activated some of its target signalings including VEGF signaling. Although transplantation of cMSCs alone did not improve LV remodeling and function, cMSCs co-transfected with c-Myc and Oct4 promoted a more positive effect in their survival and reparative properties, increased animal survival, reduced infarct size, decreased scar thickness, inhibited LV remodeling, and improved heart function 30 days after MI. Significantly, Oct4 promoted MEndoT (“Rescue me” signal) of cMSCs after both c-Myc stimulation in vitro and transplantation into the infarcted heart.

**Conclusions:**

Myocardial Isch drives resident cMSCs toward multiple phenotypes. Oct4 interacts with c-Myc to promote MEndoT capacity of cMSCs and improve their survival and reparative effects through upregulation of angiogenesis-related signaling pathways. These findings may identify novel targets for stem cell therapy.

**Supplementary Information:**

The online version contains supplementary material available at 10.1186/s13287-022-03120-7.

## Background

Although mesenchymal stem cells (MSCs) have emerged as an attractive source for cardiac cell therapy, poor survival, and potential adverse effects are major hurdles of MSCs in the treatment of myocardial infarction (MI) [1, 2]. Cardiac MSCs (cMSCs), which reside in close proximity to the site of injury, have been considered as a more suitable cell source for cardiac repair. However, cMSCs have both favorable and unfavorable effects on the infarcted myocardium [3]. cMSCs under different conditions of cardiac stress exhibit differences in differentiation potential, transcriptome, and secretome, producing their specific characteristics and functions in their application. Subject to inflammatory responses, cMSCs are able to adopt a proinflammatory or anti-inflammatory phenotype. The proinflammatory activities of MSCs during early-stage inflammation may be beneficial in mounting a proper immune response [4]. However, cMSCs in chronic cardiac ischemia (Isch) may preserve a chronic inflammatory response and exacerbate ventricular adverse remodeling and heart failure [5]. cMSCs have also been considered as one source of myofibroblasts promoting fibrosis and tissue stiffening, causing cardiac dysfunction [6]. These unfavorable outcomes are important because inflammation and fibrosis are thought to play crucial roles in cardiac remodeling and heart failure [7]. It is generally believed that mesenchymal cells can contribute to tumor angiogenesis through mesenchymal-to-endothelial transition (MEndoT) [8]. As such, further study on cMSCs-mediated MEndoT within ischemic myocardium is critical, with the hope of identifying factors and mechanisms that contribute to providing beneficial strategies for cMSC phenotypic transitions as novel therapeutic targets. Somatic cells can be reprogrammed to induced pluripotent stem cells (iPSCs) by exogenous factors, originally Oct4, Sox2, Klf4, and c-Myc [9]. Our previous study indicates that Oct4 overexpression enhanced the survival and functions of very small embryonic-like MSCs in infarcted hearts [10]. Importantly, we demonstrated that Oct4 directly triggers MSC functions. However, how Oct4 works with these other transcription factors to regulate the phenotypic and functional plasticity of cMSCs remains poorly understood.

In order to better explore the possible positive effects of cMSCs on myocardial repair, we need to determine whether and how these stem cell transcription factors affects the MEndoT of MSCs. We isolated MSCs from myocardial tissues and peripheral blood of rats within chronic myocardial Isch and defined their survivability and phenotypic properties under Isch condition. Given the superiority in angiogenic characterization, we used cMSCs to perform their comparison under different action of Oct4 overexpression or knockdown in co-transfection with other stem cell factors. Based on multiple functions of c-Myc on fibrosis and angiogenesis of MSCs within chronic myocardial Isch, we aimed to improve the survival and the MEndoT of cMSCs by co-treatment with c-Myc and Oct4 under chronic Isch. Our results could provide evidence that the cooperation of Oct4 and c-Myc may impact cMSCs phenotypic transitions important in the pathogenesis of Isch heart disease.

## Methods

An expanded description of the Materials and Methods is available in the Online Data Supplement.

### Antibodies and reagents

Additional file 6: Table S1 and Additional file 7: Table S2 list the primer sequences and the antibodies used to analyze mRNA and protein levels, respectively. 4',6-diamidino-2-phenylindole (DAPI, catalog 28,718–90-3) was purchased from Sigma-Aldrich (St. Louis, MO, USA).

### Animals

Inbred Lewis rats were used. The Animal Care and Use Committee of GuangZhou Red Cross Hospital Medical College of Ji-Nan University approved all animal experiments, which were in compliance with the Guide for the Care and Use of Laboratory Animals published by The National Academies Press (http://www.nap.edu/). Rats were weighed and anesthetized with a mixture of ketamine/xylazine (100/15 mg/kg, IP), and were euthanized by CO2 inhalation upon completion of the study. To isolate and characterize cMSCs from myocardial Isch or sham rats, we used 12-week-old, 300 g Lewis male rats as donors. To induce MI for cMSCs transplantation experiments, we used 11-week-old, 250 g Lewis female rats as recipients.

### Model of myocardial isch and sham isch

The model of myocardial Isch was established by induction of MI. MI was induced by ligating the left anterior descending coronary artery (LAD) as our previously described [10, 11]. MI was confirmed by visual blanching distal to the occlusion site and by echocardiography 24 h after MI. Animals with left ventricular (LV) ejection fraction (EF) < 70% and fractional shortening (FS) < 35% evaluated by echocardiography after induction of MI were selected. For sham Isch, the suture was passed around the coronary artery and removed without ligation. The thoracic incisions were closed with 5–0 silk sutures (Additional file 2: Fig. S1).

### Isolation, expansion, and purification of MSCs

To evaluate the effect of chronic ischemia on resident MSCs, MSCs were isolated from the heart and abdominal aortic blood of rats 30 days after MI or sham operation, and cultured via the adherent culture method, as described previously [12]. The fourth generation cells were used for subsequent experiments including their purity, viability, characteristics, pluripotency, and gene transfection, as shown in Additional file 2:Fig. S1.

### Characterization of MSCs

cMSCs and pbMSCs were defined according to the 3 criteria of the International Society for Cellular Therapy [13]: (1) adhesion to plastic, (2) expression of a specific combination of surface markers, and (3) differentiation potential (trilineage differentiation into adipocytes, osteoblasts, chondrocytes, and blood vascular cells) [10]. To define the phenotype of cMSCs and pbMSCs, we analyzed cultured cells at passage 4 for rat MSC markers by flow cytometry using the following fluorescence antibodies: SH2, SH3, CD90, CD147, CD34, CD45, and CD133. Mouse IgG1, IgG2a, and IgG2b (Becton Dickinson) were used as isotype controls, and marker expression was evaluated using FACS.

### In vitro directed differentiation of MSCs

To examine the in vitro trilineage differentiation of cMSCs and pbMSCs, we induced the MSCs to differentiate toward osteogenesis, chondrogenesis, adipogenesis, and angiogenesis by growth factor supplementation and growth on defined matrices. For osteogenesis, the MSCs were induced with an osteogenic differentiation medium kit (HUXUB-90021, Cyagen, Soochow, China). Alizarin red staining was performed to evaluate osteogenic products as previously described [14]. For chondrogenesis, the MSCs were induced using a chondrogenic differentiation medium kit (HUXUB-9004, Cyagen, Soochow, China) and evaluated by alcian blue staining [15]. For adipogenesis, the MSCs were cultured by an adipogenic differentiation medium kit (HUXUB-9004, Cyagen, Soochow, China). The formation of lipid vacuoles was assessed by Oil Red O staining [16]. For vascular differentiation, growth factors bFGF (5 ng/ml; Invitrogen), and VEGF (20 ng/ml; R&D Systems; Minneapolis, MN, USA) were added. Angiogenesis was detected using immunofluorescence with factor VIII and alpha smooth muscle actin (α-SMA) double-positive staining [10].

### Cell viability

For the cell viability assay, MSCs were seeded at 2 × 10^3^ cells/well on a 96-well plate at 37˚C and cultured for 48 h. Cell viability was assessed by visual cell counts after performing a trypan blue exclusion assay. Cell growth was measured using a cell counting kit-8 (CCK-8, Sigma) according to the manufacturer’s protocol in the different time of 0, 24 h, 48 h, 72 h, and 96 h after different treatments.

### Angiogenesis array

To define the levels of angiogenesis-related cytokine secretion from cMSCs and pbMSCs, we used an Angiogenesis Protein Array kit (QAR-ANG-100, RayBiotech), cultured MSCs for 72 h, and collected their secreted medium [17]. Concentrations of cytokines were determined by Quasys Q-View imaging and a software system. For all multiplex assays, samples were run in triplicate wells.

### Immunofluorescence

Cells or tissues were incubated with primary antibodies (Additional file 7: Table S2) and observed under a fluorescence microscope, as described in detail in Online Supplemental Data.

### Gene expression analyses

Gene expression analyses were performed by Miltenyi Biotec Genomic Services as previously described [18]. The results were viewed as a heatmap using “pheatmap” in the R package [19].

### Real-time quantitative PCR (qRT-PCR) and immunoblotting

Cultured MSCs and peri-infarct myocardial tissues from the autopsied tissues were harvested and pulverized to extract RNA or protein for qRT-PCR and immunoblotting, as described in Online Supplemental Data (Additional file 1: An expanded Materials and Methods).

### c-Myc and Oct4 transfection

Retroviral plasmid vectors, pMXs, overexpressing (*oe*) c-Myc or Oct4, were transfected with pReceiver-LV233 lentiviral vector (GeneCopoeia (Rockville, MD) into cMSCs with the Fugene HD reagent, as directed by the manufacturer’s instructions. A pSi-LVRU6GP vector with a puromycin resistance cassette (GeneCopoeia, Rockville, MD, USA) was used to express small interfering RNAs (*si*RNAs) to knock down *β*-catenin & Oct4 expression. Control siRNA duplexes were used as the control (CON).

### Cell grouping settings

In order to determine the effects of c-Myc and Oct4 in the gene expression of cMSCs, the cells were randomly divided into the following three groups according to different intervention methods: no intervention group (served as control group), c-Myc overexpression group (*oe*c-Myc), and Oct4 overexpression (*oe*Oct4); In order to determine the effects of Oct4 in cooperation with c-Myc on the phenotypic remodeling of cMSCs, the experiments were divided into: c-Myc overexpression (*oe*c-Myc) with or without Oct4 overexpression (*oe*Oct4 ( +) or without (−)), c-Myc knockdown group (*si*c-Myc ( +)) with or without Oct4 overexpression (*oe*Oct4 ( +) or without (−)). After transfection, all groups of cMSCs were cultured for 48 h under hypoxic conditions.

### Hypoxic treatment

Cells were removed and exposed to hypoxic conditions (37 °C, 93% N_2_, 5% CO_2_, and 2% O_2_) in a water-jacketed CO_2_ incubator. The hypoxic condition was maintained throughout all subsequent analyses.

### Bulk RNA-seq

Total RNA was isolated using Trizol (Invitrogen) from cMSCs isolated from Isch or Sham hearts, or cMSCs overexpressed with or without c-Myc or Oct4, and cultured for 48 h under hypoxic conditions. Bulk RNA-seq analysis was undertaken in triplicate of total RNAs (1 µg) from these three groups of cMSCs according to the manufacturer's instructions. cDNA libraries were sequenced by using the HiSeq 2500 RNA-seq platform (Illumina, San Diego, CA, USA) as 100-base pair single-end chemistry at the Australian Genome Research Facility. RNA-seq data were counted over gene exons using featureCounts 2.0.1. Genes were annotated according to the Rattus_norvegicus.Rnor_6.0.104 annotation file [20]. The DESeq2 Bioconductor R package was used to identify differentially expressed genes at a 5% false discovery rate (*P* value adjusted ≤ 0.05) by using the Benjamini–Hochberg procedure to adjust P values [21]. We used Ingenuity Pathway Analysis to perform Gene set enrichment analysis. Significantly enriched pathways were identified using a 5% false discovery rate cutoff, and their enrichment significance was quantified using − log_10_ of P value adjusted [22]. Data are expressed as pathways downregulated or upregulated in Isch cMSCs compared with Sham cMSCs, or overexpressed c-Myc or Oct4 cMSCs compared with control cMSCs. The raw counts were loaded into R 4.1.0 (R Foundation for Statistical Computing, Vienna, Austria) for statistical analysis. We used the pheatmap function to perform hierarchical clustering analyses.

### Transcriptome analyses

Gene expression was analyzed by Illumina HiSeq 2000 and as described in the Supplemental Experimental Procedures. We analyzed the genes with differential expression (FDR < 0.05) between the Sham cMSCs and the Isch cMSCs using GO-Elite (http://www.genmapp.org/go_elite/) [23]. We used Whole Genome rVISTA [24] to identify enriched predicted transcript factor (TF) binding sites among these gene sets.

### Tube formation assay

Liquid Matrigel (BD Biosciences, USA, BD Matrigel Matrix Cat. No. 356234) was added into 96-well tissue culture plates after the induction of MSCs differentiation into blood vascular cells. The in vitro tube formation assay was performed using the Matrigel (BD Bioscience, USA) according to the manufacturer's instructions. Cells were seeded into the Matrigel-coated 96-well plate, and after required treatments for 72 h of hypoxic culture, images were captured using a bright-field microscope. The observed tubes were counted [25]. Four representative fields are counted and the average of the total area of complete tubes formed by cells per unit area is compared by Image-Pro Plus®.

### Chromatin immunoprecipitation assays (CHIP)

About 2.0 × 10^6^ cells were used in each ChIP experiment. ChIP assays were performed according to manufacturer's protocol from a ChIP assay kit (Merck Millipore). The DNA samples were detected by using real-time PCR analysis. To amplify the c-Myc binding site in the Oct4 promoter, the primers sequences were as shown in Tab. S1.

### EGFP labeling

At 24 h after transfection with the *oe*c-Myc, *si*c-Myc, *oe*Oct4, *si*Oct4, or control vectors, cMSCs were co-transfected with a lentiviral vector containing enhanced GFP cDNA (EGFP), as described previously [26].

### Cell therapy

Male rats and female rats were used as donors and recipients, respectively. MI was induced in the female rats by ligating LAD. Electrocardiography (ECG) was performed to confirm MI 1 min after operation. Animals were eligible for cell therapy on the basis of ECG criteria (ST-segment elevation or left bundle-branch block). For cell transplantation experiments, we injected the male cMSCs transfected with *oe*c-Myc, *si*c-Myc, *oe*Oct4, *si*Oct4, or control vectors, or phosphate-buffered saline (PBS, 20 ul) into the ischemic border zone (5×10^6^ cells, four sites, 5 µl per site, 1–2 cm apart). Female rats with LVFS ≥ 35% at day 1 after MI were excluded from the study.

### Echocardiography

Light anesthesia was induced by inhalation of 2% isoflurane/98% O_2_ and subsequently maintained by 0.5% to 1% isoflurane. To assess LV remodeling and function after cell therapy, Echocardiography was performed using a 7.5-MHz phased-array transducer (Acuson Sequoia 256, Siemens, Mountain View, CA) at pre-MI, day 1 or 30 after MI and cell therapy as previously described [10]. LV end-diastolic volume, and internal diameter at diastolic phase (LVEDv and LVEDd, respectively) were measured using the biplane area-length method. LVFS was calculated according to the modified Simpson method: FS (%) = [(LVIDd-LVIDs)/LVIDd] × 100, where LVID is LV internal dimension, s is systole, and d is diastole. All measurements represented the mean values of the signals from three consecutive cardiac cycles and were carried out by two experienced technicians who were unaware of the identities of the respective experimental groups.

### Histopathologic evaluation

At the end of each study (30 days after cell transplantation), hearts were harvested, fixed by 4% paraformaldehyde (PFA) solution, or frozen at − 80 °C. The frozen hearts were cut transversely into 1.2-mm-thick slices and stained with 1% 2,3,5-triphenyltetrazolium chloride (TTC). Infarct and LV area were measured by automated planimetry using Image J software. The infarct size was expressed as a percentage of the total LV area.

The fixed heart samples were embedded in paraffin, and cut into 4 µm transverse sections at different levels. Hematoxylin and eosin (H&E) and Masson’s trichrome staining were performed on paraffin-embedded sections to determine extent of cardiac inflammation, fibrosis, and viable myocardium. For each section, ten to fifteen images were acquired from randomly selected fields in infarct and non-infarct areas. Images analysis was conducted using Image J software (National Institutes of Health). The severity of inflammation damage was evaluated as the percent of inflammatory cells within peri-infarct regions. Viable myocardium was calculated by multiplying myocardium density by viable myocardial volume. The percent value of viable myocardium area was estimated by image tool 3.0. Infarct size was determined by planimetric measurement with a digital image program (Scion ImageJ) and calculated by dividing the sum of the planimetered endocardial and epicardial circumferences occupied by the infarct by the sum of the total epicardial and endocardial circumferences of the LV on three transversal sections from the apex to the base. Relative scar area was computed as the ratio of nonviable to total pixels in the LV. Collagen density was calculated as the ratio of positive staining area to the total scar area.

To determine the survival of implanted male cMSCs from Isch hearts, we stained the heart sections from female recipients with antimouse sex-determining region Y protein (E-19) (Santa Cruz Biotechnology, Inc.) and hematoxylin (for nuclear staining).

To assess the effect of *c*MSCs on angiogenesis in the infarct site, heart sections were immunolabeled with anti-factor VIII (vWF) antibody. Vessel density was expressed as the number of factor VIII endothelial cells per square millimeter. Alexa Fluor 488-conjugated goat anti-rabbit IgG was also used for visualization of anti-laminin antibody to analyze capillary density (capillaries/cardiomyocyte). Cross-sectional area and numerical density of laminin-outlined transversely cut cardiac myocytes were determined in the same regions used for capillary analysis, and a capillary-to-myocyte ratio was calculated based on the numerical densities calculated for capillaries and cardiac myocytes, as previously detailed [27]. A pathologist who was blinded to group identity evaluated the capillary density and cell count by counting vessels and cells in the chosen areas.

### Statistical analysis

Data are presented as the mean ± standard error of the mean (SEM). Discrete variables are presented as frequency and proportion. By performing normality test (Shapiro–Wilk test) and homogeneity test of variance, the data that satisfy normal distribution and equal variance assumptions were used for one-way ANOVA analysis of these variables. When the data were conferred for normal distribution but non-homogeneity of variance, Welch ANOVA analyses were performed. Comparisons were performed using the *x*^2^ or Fisher’s exact test for discrete variables. A 95% confidence interval (CI) (*p* < 0.05) was considered significant.

## Results

### Myocardial ischemia alters the phenotypes and secretome MSCs

MSCs are multipotent tissue-resident cells, and exhibit a selective ability for tissue repair by engrafting and differentiating into desired cells at the damaged tissues [28–30]. To determine the effect of myocardial Isch on resident cardiac MSCs (cMSCs) and peripheral blood MSCs (pbMSCs), we isolated MSCs from male Lewis rats with Isch 30 days after MI (*n* = 10) or sham operation (*n* = 10) as previously described [10, 31]. Rats which were not ligated in the same part of the hearts and served as sham group (Sham). Both cMSCs and pbMSCs, isolated after either MI or sham operation, were plastic-adherent, showed universe spindle like morphology, and had good clonogenicity (Additional file 3: Fig. S2A). We next evaluated cell proliferation rate for 96 h and found that cMSCs from the ischemic hearts had the highest growth rate compared with other groups (Additional file 3: Fig. S2B). Immunophenotypic analyses by flow cytometry indicated that compared with other MSCs, the Isch cMSCs were strongly positive for mesenchymal lineage markers SH2, SH3, and CD147, while negative for hematopoietic lineage markers CD 34, CD45, and CD117 (Additional file 3: Fig. S2C). Notably, Thy-1 (CD90), which mediates fibroblast adhesion and migration [32], was expressed at lower frequency in Isch cMSCs compared to other MSCs. Last, we performed direct differentiation toward adipocyte, chondrocyte, osteoblast, and vascular cells by growth factor supplementation and growth on defined matrices. Multi-lineage differentiation into osteoblasts, adipocytes, chondrocytes, and blood endothelial cells confirmed the pluripotency of the MSCs (Additional file 3: Fig. S2D–G). These results suggest that myocardial ischemia affects the phenotype of MSCs, particularly cardiac MSCs.

**Fig. 1:**
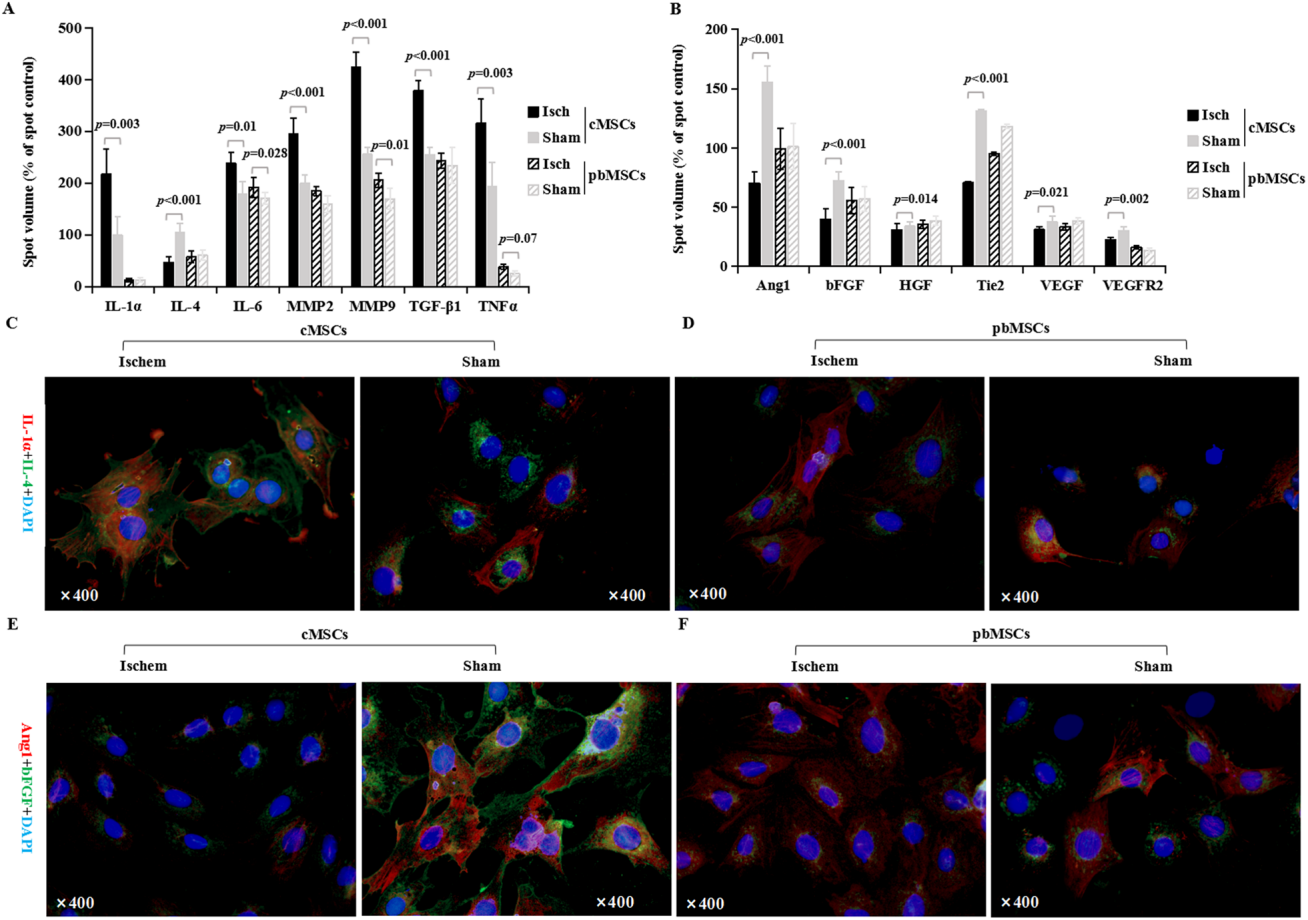
The effects of myocardial ischemia on the secretion profile of cMSCs. Rat angiogenesis arrays were utilized in order to analyze supernatants after a 48 h incubation to evaluate the effect of myocardial ischemia on MSC cytokine profiles in the conditioned medium collected from the cells post-MI and compared them with their sham counterparts. cMSCs from ischemic rats significantly changed their paracrine profile, compared with their sham counterparts. Ischem cMSCs became more inflammatory with decreased anti-inflammatory and proangiogenic factors (**A** and **B**). Results are presented as the percent of spot density to control ± SEM. Independent samples t test was used (*n* = 5 per group). (**C**–**F**) Direct fluorescence staining with IL-1*α*/IL-4 (**C** and **D**) and Ang1/bFGF (**E** and **F**) in MSCs derived from ischemic rats, compared with sham-operated rats, and counterstained with DAPI. DAPI, 4’, 6-diamidino-2-phenylindole

MSCs modulate myocardial repair through their paracrine, angiogenesis, anti-fibroblasts, and antioxidation properties after MI [33]. For that purpose, we used a rat angiogenesis array to measure the expression of 60 angiogenesis-related cytokines from the conditioned medium of cultured MSCs. Significantly, cMSCs isolated from Isch hearts secreted higher levels (1.3–2.2-fold) of proinflammatory cytokines, interleukin (IL)-1*α*, IL-6, matrix metalloproteinase 2 (MMP2) and MMP9, transforming growth factor-*β*1 (TGF-*β*1), and tumor necrosis factor-*α* (TNF*α*), compared with their Sham counterparts. Conversely, we found cMSCs from Isch hearts showed a much smaller release of anti-inflammatory factor IL-4 than those from Sham hearts (Fig. 1A). Importantly, we found that the levels of the proangiogenic factors, angiopoietin1 (Ang1), basic fibroblast growth factor (bFGF), hepatocyte growth factor (HGF), vascular endothelial growth factor (VEGF), tunica interna endothelial cell receptor 2 (Tie2), and vascular endothelial growth factor receptor2 (VEGFR2), were significantly decreased in cMSCs from Isch hearts (1.1–2.2-fold lower) compared with Sham cMSCs (Fig. 1B). It is notable that myocardial Isch induced a different secretory profile in pbMSCs. Although the levels of IL-6, MMP9, and TNFα were slightly increased in pbMSCs from Isch rats (about 1.2-fold higher) compared with pbMSCs from Sham rats, most cytokines secreted from Isch pbMSCs, such as IL-1*α*, IL-4, TGF-*β*1, MMP2, Ang1, bFGF, HGF, Tie2, VEGF, and VEGFR2, showed no change in cytokine levels, compared with Sham pbMSCs. Similarly, immunofluorescence results reveled that higher expression of IL-1*α* was related with lower level of IL-4, Ang1, and bFGF in Isch cMSCs (Fig. 1C–F). These results indicate that myocardial Isch induced cMSCs remodeling by decreasing of proangiogenic and anti-inflammatory factors.

### Ischemia mediated imbalance in expression of c-Myc and Oct4 in cMSCs

**Fig. 2:**
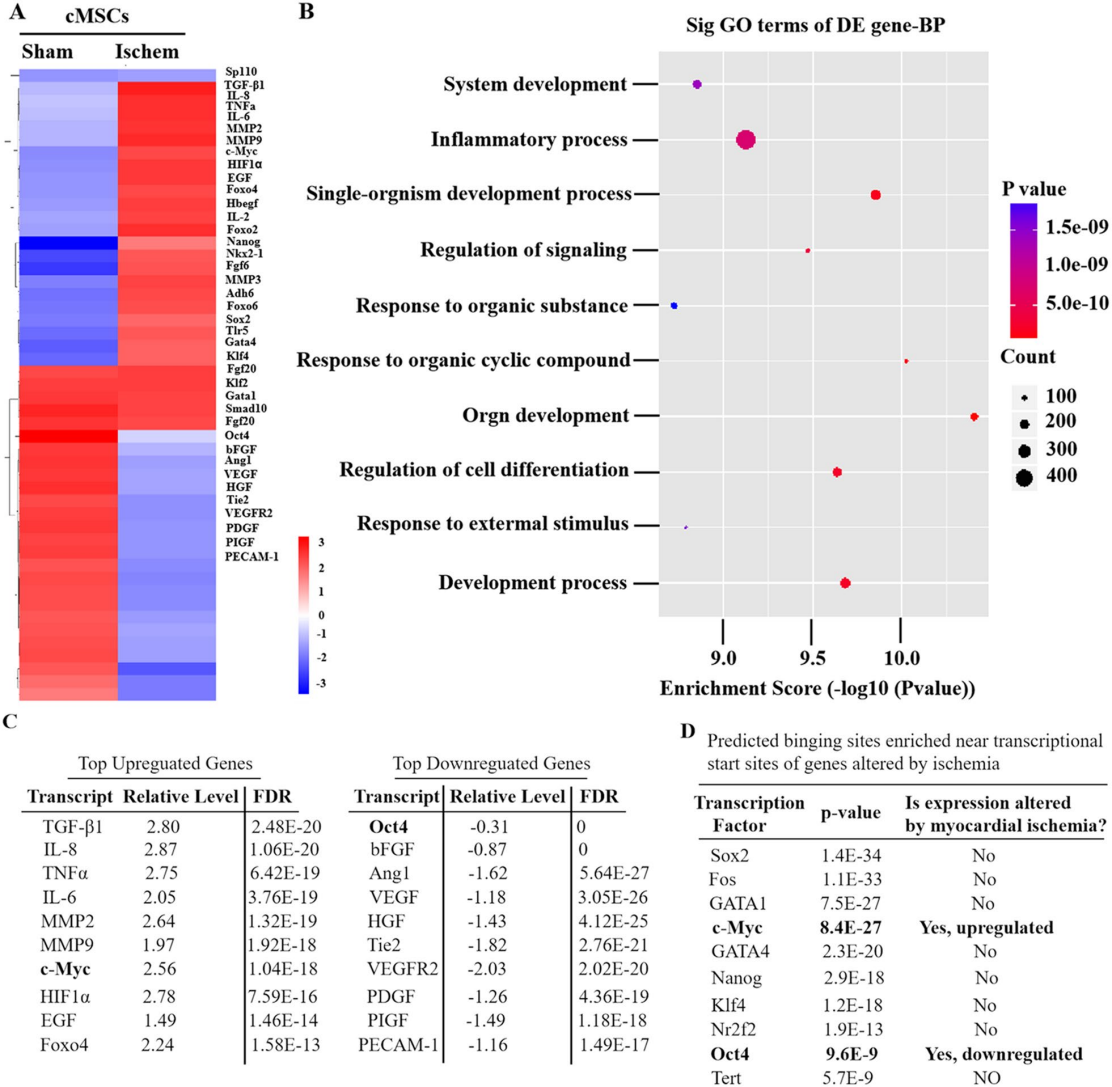

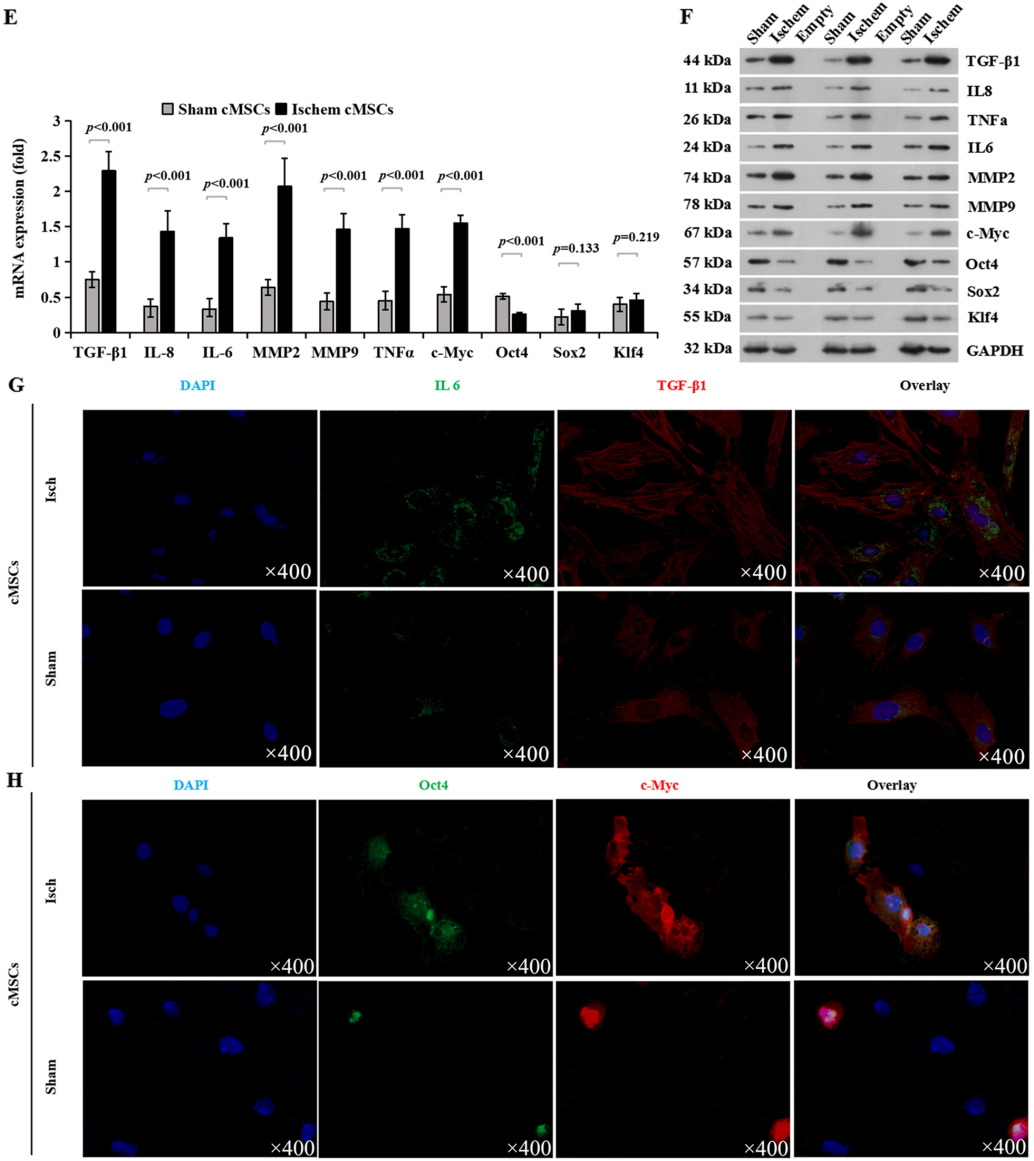
Hypoxia-Induced Imbalance Expression of Transcriptome Factors in cMSCs. **A** Transcriptome gene expression analysis of ischemic cMSCs and sham cMSCs. Heatmap of shared upregulated or downregulated genes with logarithmic fold change. Downregulated genes are represented in blue, and upregulated genes are represented in red. **B** Dotplot of top 10 enriched GO terms. **C** List of the most significantly upregulated (left) and downregulated (right) transcripts upon ischemia assayed by RNA-seq. **D** List of the top TFs with enriched predicted binding sites among genes altered upon ischemia. Among these TFs, c-Myc and Oct4 had altered expression after induction of myocardial ischemia. **E** and **F** The mRNA and protein expression levels of these transcriptome factors were verified in both cMSCs using qRT-PCR **E** and immunoblotting **F**. All data are the means ± SEM. *p* < 0.05: * *vs*. sham (*n* = 10 per group), independent samples *t* test was used. (**G** and **H**) Different expression of inflammatory or angiogenic factors in cMSCs from the ischemic or sham hearts by immunofluorescence staining. Positive staining for IL6 (green) and TGF-*β*1 (red) indicates higher inflammation in ischemic cMSCs compared with sham cMSCs **G**. Lower expression of Oct4 (green) and higher expression of c-Myc (green) in ischemic cMSCs compared with sham cMSCs indicates ischemia inducing imbalance expression of transcriptome factors **H**. DAPI is used to counterstain the nucleus (blue). Representative images of at least 3 different cells are shown. cMSCs indicate cardiac mesenchymal stromal cells. DAPI, 4’, 6-diamidino-2-phenylindole

Since myocardial ischemia switched cMSCs toward an inflammatory phenotype, we searched for putative transcription factors (TFs) involved in inflammatory transformation of cMSCs. We first examined genome-wide transcriptome changes by RNA-seq. Expression of over 500 genes was altered (**Fig. ****2****A**). We further narrowed the screening and screened out the top ten upregulated genes (TGF-β1, interleukin-8 (IL-8), TNF*α*, IL-6, MMP2, MMP9, c-Myc, HIF1*α*, EGF, and Foxo4) and downregulated (Oct4, bFGF, Ang1, VEGF, HGF, Tie2, VEGFR2, PDGF, PIGF, PECAM-1) as the hub genes (Fig. 2C). Gene ontology (GO) analysis suggested that ischemia regulated inflammation, development, and differentiation (Fig. 2B). We hypothesized that ischemia may promote inflammation transformation by regulating stem cell transcription factors. To test this, we used Whole Genome rVISTA [24] to search for predicted TF binding sites that are enriched within the set of genes regulated by ischemia. Two TFs, c-Myc and Oct4, stood out, as their transcripts were also among those most obvious changes upon ischemia (Fig. 2C, D). As c-Myc is one transcription factor of the four Yamanaka factors involved in proliferation, apoptosis, differentiation, immunity, and somatic cell transformation [34]. As for other three Yamanaka factors, Oct4 decreased significantly in Isch cMSCs, while other two factors, Klf4 and Sox2, showed no significant difference between Isch cMSCs and Sham cMSCs (Fig. 2A). To ascertain the expression difference, we performed qRT-PCR and western blotting to detect the mRNA and protein levels of these TFs. Myocardial ischemia significantly increased the mRNA and protein expressions of IL-6, IL-8, TNF*α*, TGF-*β*1, MMP2, MMP9, and c-Myc in cMSCs. Nevertheless, the expression of Oct4 was much lower in Isch cMSCs than in Sham MSCs (Fig. 2E, F). This expression difference was further confirmed by immunofluorescence staining. Compared with Sham cMSCs, Isch cMSCs showed a significant enrichment of IL-6 and TGF-*β*1 (Fig. 2G), higher expression of c-Myc, and lower expression of Oct4 (Fig. 2H). Importantly, c-Myc is distributed in both the cytoplasm and nucleus of Isch cMSCs, but mainly located in the nuclei of Sham cMSCs. Oct4 accumulated mainly in the nuclei of both Isch cMSCs and Sham cMSCs (Fig. 2H). Together, our results indicate imbalance in expression of Oct4 and c-Myc under ischemic condition regulating genes associated with remodeling of cMSCs. Therefore, to explore the mechanisms involved in cMSCs remodeling under hypoxic or ischemic conditions, we focused on Oct4 and c-Myc in cMSCs from the ischemic hearts.

### c-Myc and Oct4 show divergent phenotypes of cMSCs under hypoxia

Both Oct4 and c-Myc regulate pluripotency and stemness of adult stem cells [35, 36], and may regulate genes associated with angiogenesis and inflammatory post-MI [10, 37]. Therefore, we investigated their roles in cMSCs remodeling in vitro, and cultured Isch cMSCs transfected with a lentivirus encoding overexpressed (*oe*) c-Myc (*oe*c-Myc), Oct4 (*oe*Oct4), or vehicle (served as control, CON) for 48 h under hypoxic conditions. Normoxic cultivation of cMSCs with or without transfection of c-Myc or Oct4 was also performed to clarify these effects under normoxia. We confirmed that c-Myc and Oct4 transfection was efficiently induced in cMSCs under normoxia, and hypoxia further increased their expression (Additional file 4: Fig. S3A, B). Notably, dynamic analysis of the percentage of BrdU-positive cells/total number of cells revealed that *oe*c-Myc stimulated cell proliferation kinetically, and *oe*Oct4 further enhanced this proliferation (Additional file 4: Fig. S3C, D). Similar to BrdU expression, immunofluorescence showed that the level of Ki67 expression was higher in cMSCs receiving overexpression of c-Myc or Oct4 compared with their respective controls (Additional file 4: Fig. S3E, F; Additional file 4: Fig. S3I, and J). In line with these findings, CCK-8 assay in optical density (OD) value, which was a quantitative index for the growth capacity, showed the same trend in transfection efficiency as observed for the generation of cell proliferation (Additional file 4: Fig. S3G, H). Importantly, compared with normoxia, the enhancement of this index was remarkably observed in the *oe*Oct4 treating cMSCs after 48-h of hypoxic culture. Together, these results suggest that the transfection of c-Myc or Oct4 favors the growth of cMSCs, especially under hypoxic conditions. Thus, we next focused on evaluating the effects of c-Myc and Oct4 overexpression on the phenotype change of cMSCs under hypoxia.

**Fig. 3:**
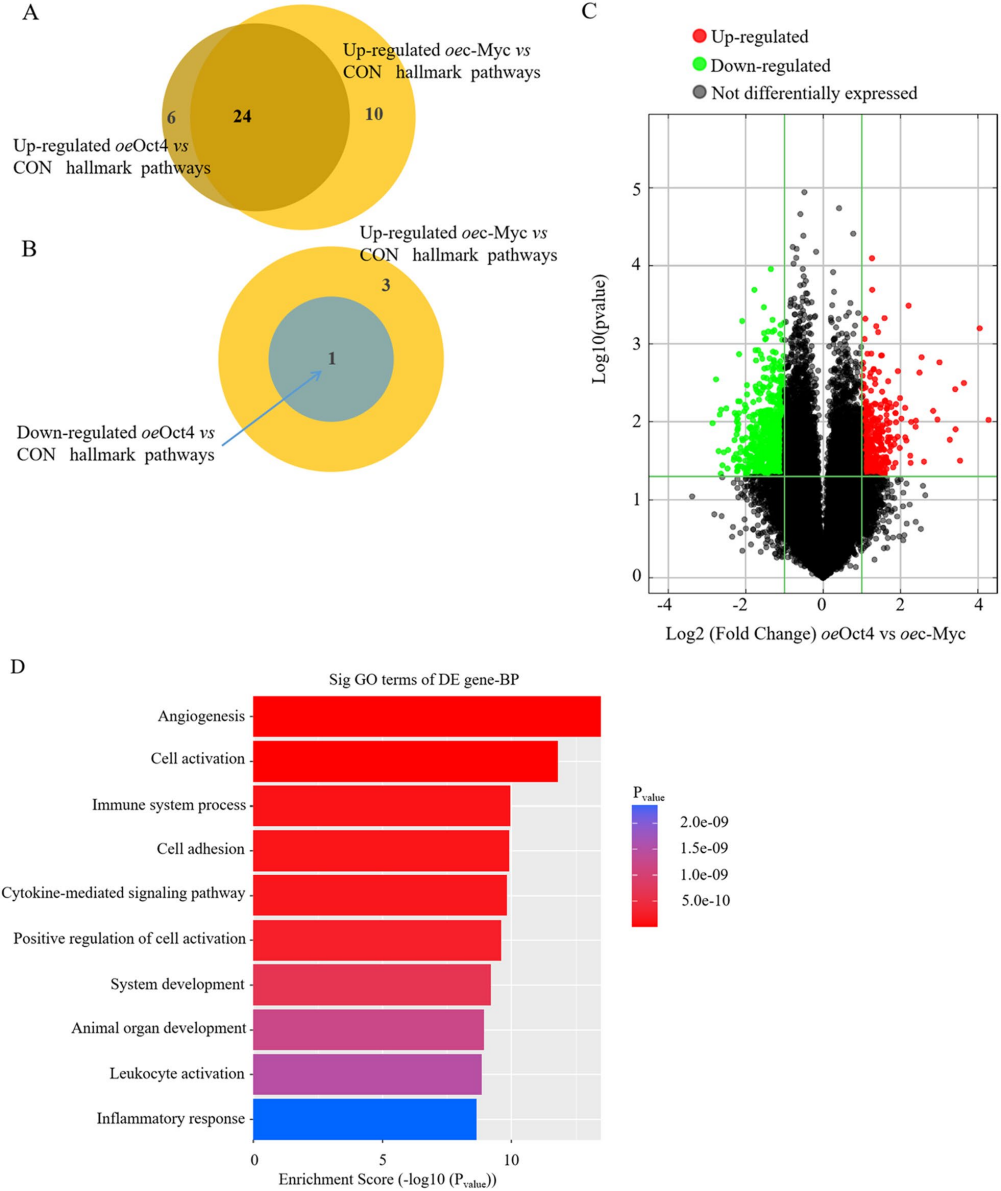

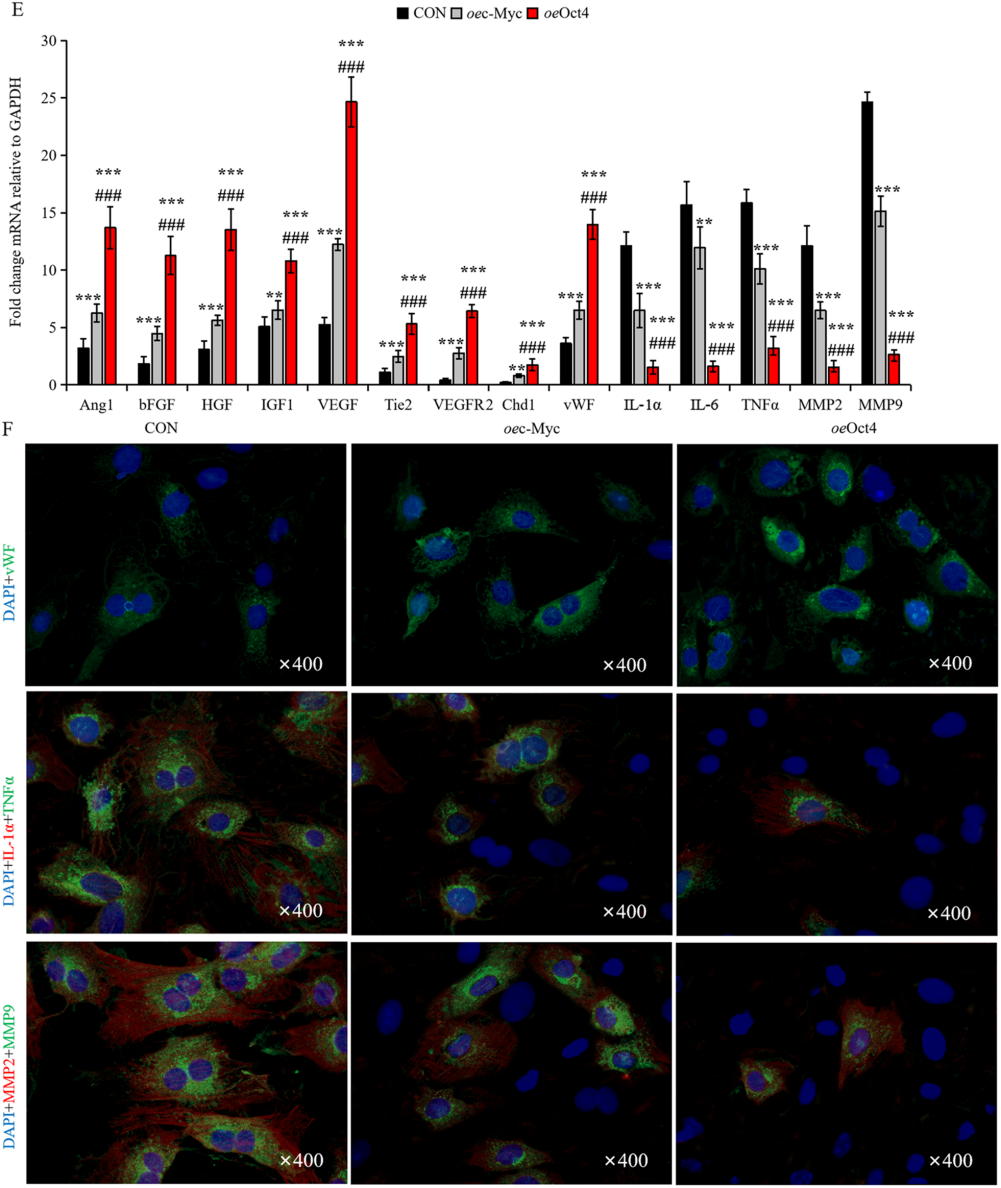
c-Myc/Oct4 induces divergent cellular angiogenesis, inflammation, and fibrosis. A Venn diagram showing the common pathways between upregulated cMSCs^*oe*c−Myc^ *vs* cMSCs^CON^ and upregulated in cMSCs^*oe*Oct4^ *vs* cMSCs^CON^. **B** Venn diagram showing the common pathways between upregulated cMSCs^*oe*c−Myc^ *vs* cMSCs^CON^ and downregulated in cMSCs^*oe*Oct4^ vs cMSCs^CON^. **C** Scatter plot analysis of hallmark genes in cMSCs^*oe*Oct4^ and cMSCs^*oe*c−Myc^. Green, black, and red indicate downregulated genes, relatively stable with less than two fold change, and upregulated genes by more than two folds in cMSCs^*oe*Oct4^ compared to cMSCs^*oe*c−Myc^, respectively. **D** Bar graph showing the top 10 common pathways from **A** and all common pathways in **B** differentially regulated pathways between cMSCs^*oe*c−Myc^ *vs* cMSCs^CON^ and cMSCs^*oe*Oct4^ *vs* cMSCs^CON^ analyses. **E** mRNA levels of the indicated factors in cMSCs transfected with control vehicle, *oe*c-Myc, or *oe*Oct4. Values are relative to GAPDH. All data are the means ± SEM; statistical significance was evaluated using the unpaired two-tailed Student’s *t* test with Welch’s correction. Comparison of each genotype with its own control is indicated as follows: ^**^P < 0.001, ^***^P < 0.001. Comparison of *oe*c-Myc is indicated in the same manner but using the symbol “#”; ^##^*P* < 0.001, ^###^*P* < 0.001. **F** These cMSCs were examined via immunofluorescence for the expression of vascular endothelial marker vWF (green), inflammatory cell markers, IL-1α (red) and TNFα (green), and fibroblast markers, MMP2 (red) and MMP9 (green). Also shown is DAPI staining (nuclei; blue).

First, we investigated global changes in gene expression that accompanied angiogenic/ inflammatory phenotypes of c-Myc and Oct4 overexpression in cMSCs, and conducted bulk RNA-seq on cMSCs overexpressed with or without c-Myc or Oct4. Consistent with the above data that c-Myc and Oct4 overexpression have different effects on the growth of cMSCs, gene set enrichment analysis of bulk RNA-seq data using the MSigDb showed about 75% of the upregulated hallmark and Kyoto Encyclopedia of Genes and Genomes pathways in cMSCs^*oe*c−Myc^ *versus* cMSCs^CON^ were also upregulated in cMSCs^*oe*Oct4^ *versus* cMSCs^CON^, and nearly 25% of all upregulated pathways in cMSCs^*oe*c−Myc^ *versus* cMSCs^CON^ were downregulated in the cMSCs^*oe*Oct4^ *versus* cMSCs^CON^ (Fig. 3A, B). Then, we calculated the Log2 FC of cMSCs^*oe*Oct4^ compared to cMSCs^*oe*c−Myc^. 405 genes were upregulated more than 1 Log2 FC and 712 genes downregulated less than − 1 Log 2 FC; in total, 4.2% of genes (Fig. 3C). According to GO terms, upregulated genes in cMSCs^*oe*Oct4^ included response to angiogenesis, cell activation, immune system process, cell adhesion, cytokine-mediated signaling pathway, and positive regulation of cell activation, and the genes involved with inflammatory response were downregulated in cMSCs^*oe*Oct4^(Fig. 3D). From these signaling systems, of note are the following genes: Ang-1, bFGF, HGF, IGF-1 (Insulin-like growth factor-1), Tie2, and VEGF. These genes are all implicated in angiogenesis and MEndoT [11]. Other notable genes are vWF, VEGFR2, and Chd1 (chromatin remodeling factor 1). The majority of these genes play roles in proliferation, pluripotency, and angiogenic properties of stem cells [38, 39], while vWF is a gene encoding endothelial cell surface. By contrast, Oct4 overexpression dramatically reduced the mRNA expression levels of the inflammatory factors IL-1*α*, IL-6, and TNF*α*, and the fibrosis-related factors MMP2 and MMP9. However, compared with Oct4 overexpression, c-Myc overexpression caused a lower degree of decrease (Fig. 3E). These trends (*oe*Oct4 > *oe*c-Myc) were also confirmed by immunofluorescence which revealed more vWF^+^ cells and less IL-1^+^TNF*α*^+^ cells and MMP2^+^MMP9^+^ cells in *oe*Oct4 *versus* CON, but relatively lower levels of vWF and higher levels of inflammation and fibrosis in cMSCs^*oe*c−Myc^ *versus* cMSCs^*oe*Oct4^ (Fig. 3F). Thus, c-Myc triggers diverse cellular outcomes: hypoxia-induced cellular angiogenesis, and cellular inflammation and fibrosis, whereas Oct4 overexpression promotes angiogenesis in coexistence with inhibition of inflammation and fibrosis.

### Oct4 cooperates with c-Myc to accelerate cMSCs remodeling

**Fig. 4:**
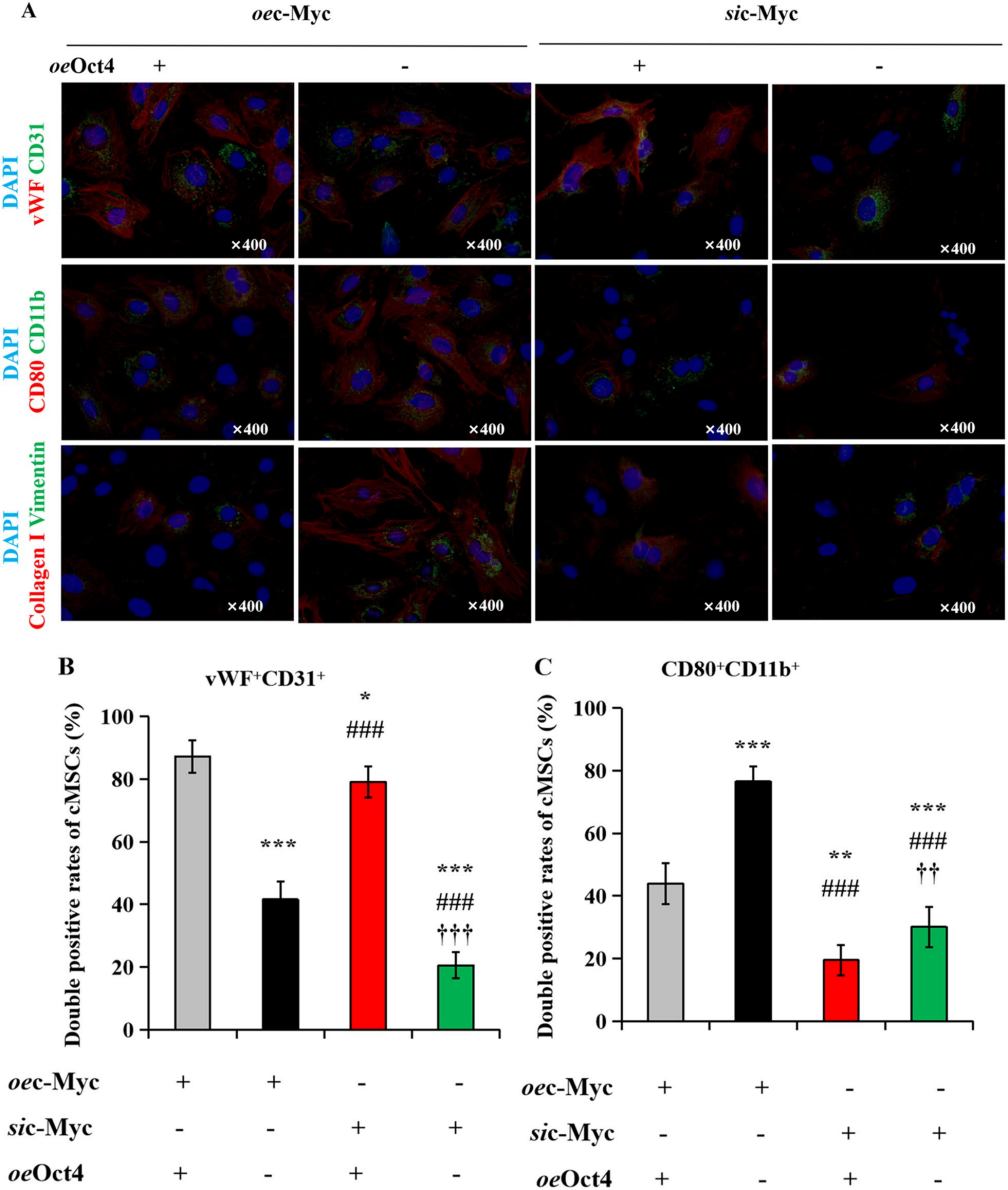

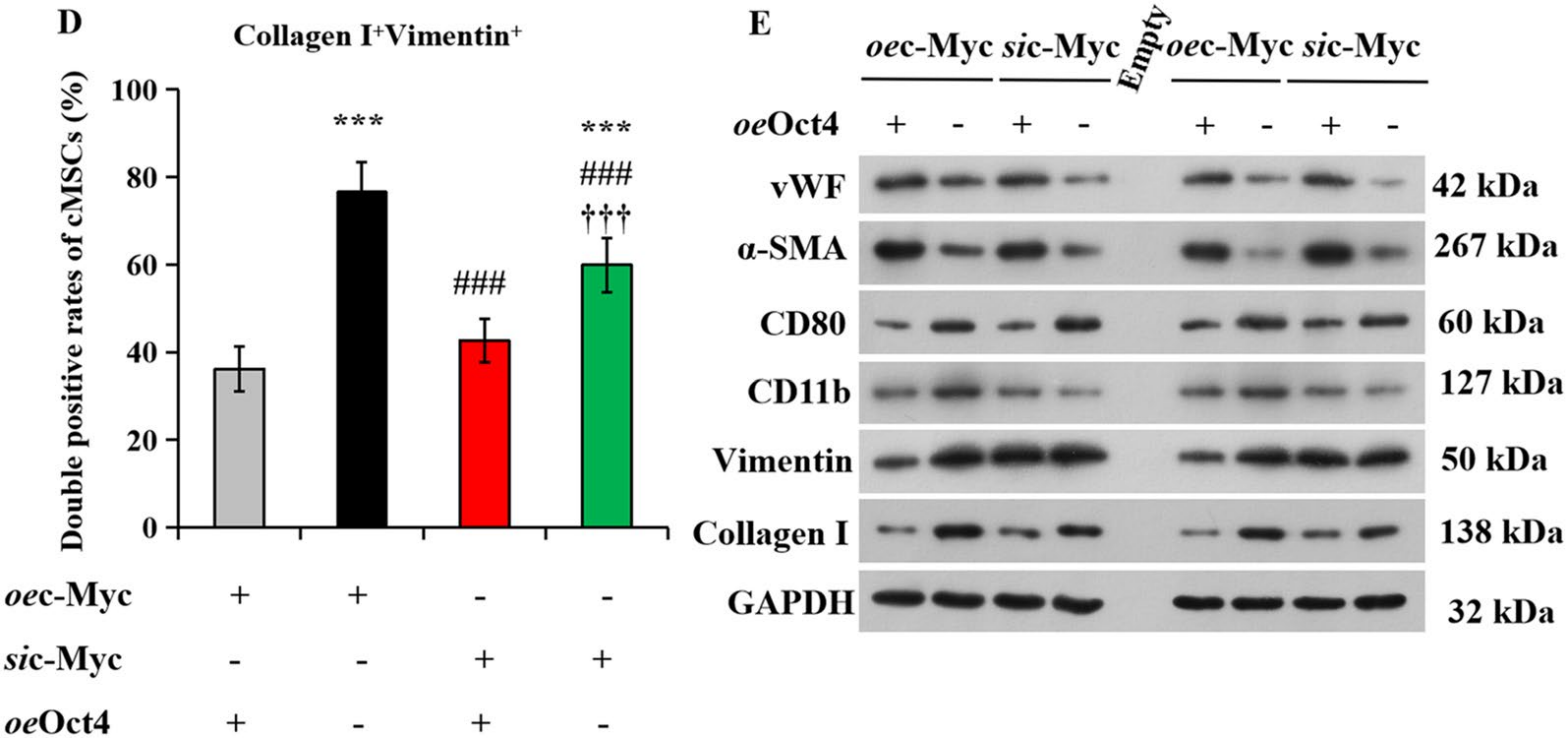
Oct4 accelerates cMSCs to switch toward an angiogenesis phenotype. **A** Angiogenesis, inflammation, and fibrosis of cMSCs subjected to transfection of *oe*c-Myc or *si*c-Myc in the absence or presence of *oe*Oct4, as determined using immunofluorescence with anti-vWF (red)/CD31 (green), CD80 (red)/CD11b (green), and collagen I (red)/vimentin (green), respectively. The nuclei were counterstained with DAPI (blue). **B** **C** **D** Double-positive cells in angiogenesis, inflammatory, or fibrosis proteins were expressed as a percentage of vWF^+^CD31^+^ **B**, CD80^+^CD11b^+^ **C**, or collagen I^+^vimentin^+^ **D** relative to all cMSCs from **A**. All data are the means ± SEM; statistical significance was evaluated using the unpaired two-tailed Student’s *t* test with Welch’s correction. Comparison of *oe*Oct4^+^*oe*c-Myc^+^ is indicated as follows: ^*^P < 0.05, ^**^P < 0.001, ^***^P < 0.001. Comparison of *oe*c-Myc or *si*c-Myc + *oe*Oct4 is indicated in the same manner but using the symbol “#,” or “†,” respectively; ^###^*P* < 0.001; ^††^*P* < 0.001, ^†††^*P* < 0.001. **E** Immunoblotting with anti-vWF/CD31, CD80/CD11b, and collagen I/vimentin in cMSCs culture system with transfection of *oe*c-Myc or *si*c-Myc in the absence or presence of *oe*Oct4. DAPI, 4′, 6-diamidino-2-phenylindole

Oct4 and c-Myc are both stem cell regulators, and play key roles in sustaining and amplifying pluripotent stem cells [40], however, the functional interaction has proven obscure. Therefore, we next investigated whether Oct4 interacts with c-Myc in vitro by simultaneous transfection of cMSCs with RNA constructs encoding Oct4 and c-Myc, or c-Myc siRNA knockdown (*si*c-Myc), or vehicle (−), seeded on Matrigel to facilitate angiogenesis, and subjected to serum starvation under hypoxia. After 72 h of hypoxic cultivation, cMSCs were collected, and their angiogenesis, inflammatory, fibroblast were analyzed by immunofluorescence and western blotting. Immunofluorescence showed the highest expressions of the vascular characteristic markers, factor VIII and CD31 in the cMSCs co-transfected with Oct4 and c-Myc, followed by that in the *si*c-Myc-transfected cMSCs co-treated with *oe*Oct4, and the lowest was observed in the *si*c-Myc-transfected MSCs (Fig. 4A, B). Conversely, Oct4 overexpression dramatically decreased the protein levels of inflammatory markers, CD80 and CD11b, and fibroblast markers including collagen I and vimentin, in *oe*c-Myc-treated cMSCs, whereas the levels of these marker proteins were significantly higher in *oe*c-Myc-transfected cMSCs than in *si*c-Myc-transfected cMSCs (Fig. 4A-D). Western blot showed similar results after Oct4 overexpression. Upregulating Oct4 in *oe*c-Myc-transfected cMSCs promoted increase in factor VIII and α-SMA expressions, and downregulated the expression of CD80, CD11b, collagen I, and vimentin. Moreover, Oct4 overexpression inhibited decrease in angiogenesis but promoted reduction of inflammatory and fibroblast in *si*c-Myc-transfected cMSCs (Fig. 4E). Overall, Oct4 accelerates cMSCs to switch toward an angiogenesis phenotype, and reduced their inflammatory and fibrosis induced by c-Myc overexpression.

### Oct4 regulates c-Myc function and translocation

**Fig. 5:**
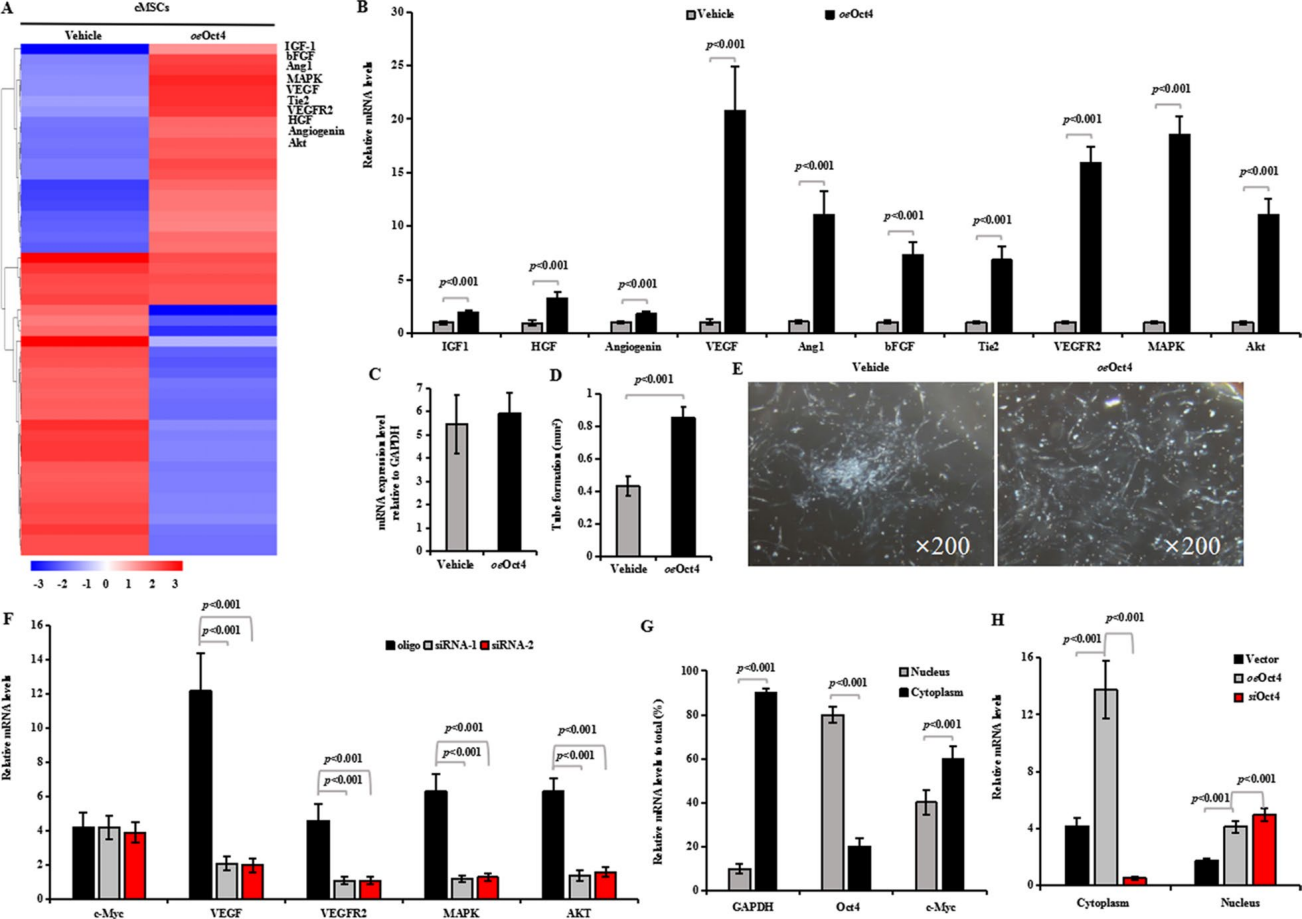

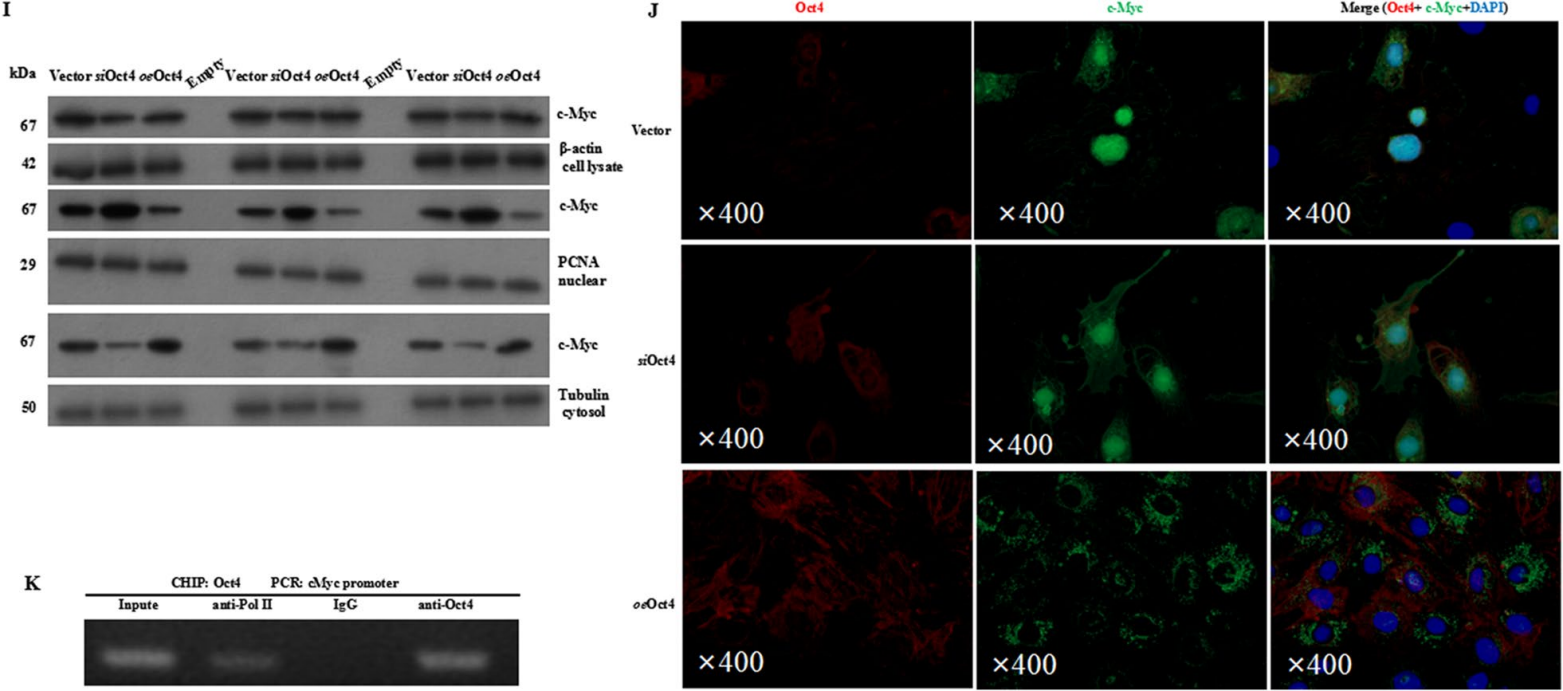
Oct4 promoted cytoplasmic translocation of c-Myc. **A** Heatmap of c-Myc-differentially expressed genes (DEGs, change relative to the mean) in vector-transfected cMSCs and Oct4-overexpressed cMSCs. The top 10 highly expressed proangiogenic factors are listed. Heatmap colors indicate directionality (red: increased; blue: decreased). **B** RNAs extracted from vector-transfected cMSCs and Oct4-overexpressed cMSCs were subjected to real-time RT-PCR. Significantly higher levels of c-Myc target factors were detected in the Oct4-overexpressed cMSCs than in the vector-transfected cMSCs. **C** The c-Myc null cMSCs were transfected with Oct4 or a control vector followed by real-time RT-PCR to confirm no significant difference in the expression of c-Myc. **D** Tube formation ability of cMSCs transfected with Oct4 or control vector. All data are the means ± SEM; statistical significance was evaluated using the unpaired two-tailed Student’s *t* test with Welch’s correction. **E** Typical images of capillary-like net-work formation of cMSCs from these two groups. **F** The siRNA-transfected cells expressed significantly lower levels of VEGF signaling signals than the control. All data are the means ± SEM, and independent samples t test was used. **G** The mRNA expression of Oct4 and c-Myc in the cytoplasm and the nuclei of c-Myc null cMSCs were detected by real-time RT-PCR. GAPDH was served as positive control. GAPDH and c-Myc were mainly expressed in the cytoplasm, while significantly higher levels of Oct4 were detected in the nuclei than in the cytoplasm. *P* value for the difference between groups was calculated by the nonparametric Kruskal–Wallis test followed by a Dunn multiple comparisons test. **H**, RNAs extracted from cytoplasm and nuclei of cMSCs transfected with Oct4, Oct4 siRNA, or the vector were subjected to real-time RT-PCR. Significantly higher levels of c-Myc were detected in the cytoplasm than in the nuclei. All data are the means ± SEM, and independent samples *t* test was used (*n* = 10, each group). **I** Western blotting revealed similar protein levels of c-Myc expression in the cell lysates. *oe*Oct4-transfected cMSCs showed more expression of cytoplasmic c-Myc than vector-transfected cMSCs, but Oct4 siRNA reversed this trend of expression, disclosing the dense expression of this factor in the nuclei. **K** Chromatin immunoprecipitation (ChIP) assay for the binding of Oct4 to c-Myc promoter. Anti-IgG was used as a negative control, anti-RNA-polymerase II was used as a positive control

We next investigated the underlying molecular mechanism responsible for the promotion of Oct4 on c-Myc-induced angiogenesis. It has been reported that in a mouse lung model of KRas^G12D^-driven adenomas, co-activation of Myc and ras can drive tumor cell proliferation by programming inflammation and angiogenesis [41]. The global transcriptional regulations were examined in the cMSCs transfected with vehicle or *oe*Oct4 after 72 h of hypoxic culture. We mined the expression of some known c-Myc targets in these cells and found that *oe*Oct4-transfected cMSCs have higher expression of angiogenesis-related signaling (Fig. 5A), which is critical in promoting c-Myc-induced angiogenesis [42]. This observation was confirmed by qRT-PCR (Fig. 5B). Especially, VEGF signaling including VEGF, VEGFR2, MAPK, and Akt [43] were specifically and significantly activated in *oe*Oct4-transfected cMSCs. To validate the effect of Oct4 in mediating c-Myc functions, we transfected the *oe*c-Myc-treated cells with either Oct4 or a control vector. Although Oct4 overexpression did not affect c-Myc mRNA levels (Fig. 5C), it promoted tube-forming ability in the c-Myc-treated cMSCs (Fig. 5D, E), confirming the role of Oct4 in mediating c-Myc-induced angiogenesis of cMSCs in vitro. To validate these results, we performed silencing assays. In contrast with Oct4 overexpression in cMSCs, silencing Oct4 markedly downregulated c-Myc targets mainly including VEGF, VEGFR2, MAPK, and AKT, compared with the control oligo, resulting in no significant change in c-Myc expression (Fig. 5F). It has been reported that altering Oct4 protein level affects its translocation from nuclear to cytoplasm [44]. We isolated total RNA from cytoplasm and nuclei and measured the levels of Oct4. For cMSCs without Oct4 overexpression, higher levels of Oct4 were detected in nuclei than in cytoplasm, but c-Myc expressed more significantly in cytoplasm than in nuclei (Fig. 5G). For cMSCs transfected with Oct4, significantly higher levels of the ectopic Oct4 were detected in the cytoplasm relative to the nuclei (Fig. 5H). Western blotting using fractionated samples showed a similar distribution of c-Myc: Silencing Oct4 resulted in higher expression of c-Myc in the nuclei than in the cytoplasm, whereas overexpressing Oct4 produced an opposite pattern of c-Myc distribution (Fig. 5I). By confocal microscopy, we detected mainly cytoplasmic c-Myc in the Oct4-transfected cells and nuclear distribution of c-Myc in the vector-transfected cells (Fig. 5J). To confirm the binding of the c-Myc promoter by Oct4, a chromatin immunoprecipitation (ChIP) assay in cMSCs was performed. It was suggested that c-Myc promoter fragments could only be amplified from chromatin precipitated with Oct4 antibodies (Fig. 5K). Taken together, our study showed that Oct4 overexpression induced cytoplasmic translocation of c-Myc. It has been reported that nuclear c-myc is readily degraded [45]. In our study, c-Myc was mainly translocated to the cytoplasm in *oe*Oct4-treated cMSCs, but we did not detect decreased level of total c-Myc. This suggested that c-Myc was colocalized with Oct4, preventing degradation (Additional file 5: Fig. S4).

### Expression of Oct4 activating c-Myc in cMSCs improves their myocardial repair

**Fig. 6:**
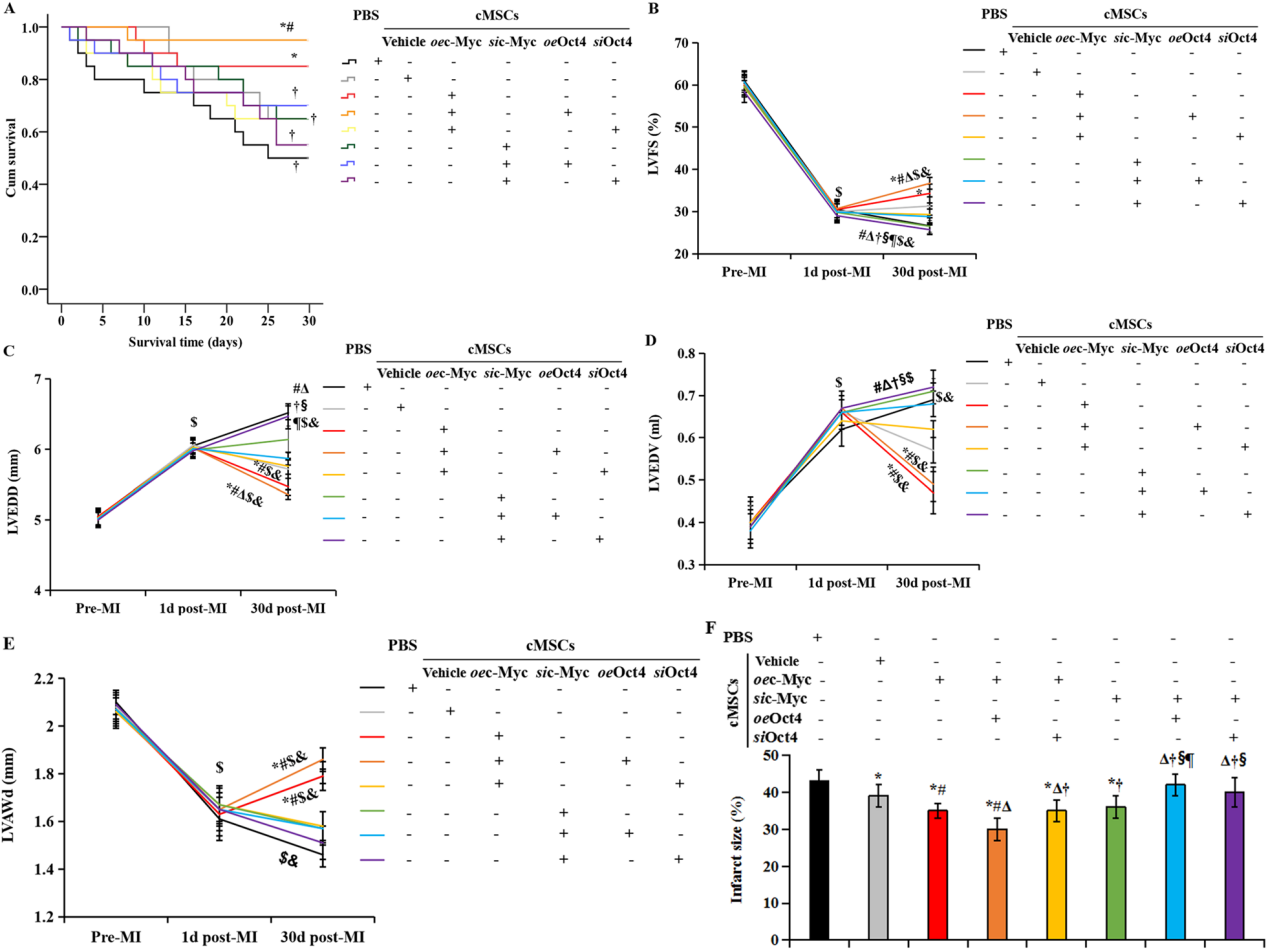
Overexpression of Oct4 and c-Myc in implanted cMSCs improves their myocardial repair in recipient rats 30 days after MI. **A** Kaplan–Meier survival rates. **B–E** Echocardiography of LVFS **B**, LVEDD **C**, LVEDV **D**, and LVAWd **E** before MI (pre-MI), 1d after MI (1d post-MI), and 30d after MI (30d post-MI). Transplantation of cMSCs with co-transfection c-Myc and Oct4 had higher survival rates and significant improvement in cardiac function and structural remodeling. However, this was canceled by inhibition of c-Myc or Oct4, which was not rescued by combining with Oct4 or c-Myc transfection. (**F**) Quantitation of infarct size as determined by TTC staining at 30d post-MI. All graphical data are the means ± SEM. Welch ANOVA analyses were performed. *p* < 0.05: ^*^vs PBS injection*,* ^#^*vs*. cMSCs therapy alone, ^Δ^*vs*. Transplantation of *oe*c-Myc transfected cMSCs, ^†^*vs*. Transplantation of cMSCs transfected with *oe*c-Myc plus *oe*Oct4, ^§^*vs*. Transplantation of cMSCs transfected with *oe*c-Myc plus *si*Oct4, ^║^vs. Transplantation of cMSCs transfected with *si*c-Myc, ^¶^vs. Transplantation of cMSCs transfected with *si*c-Myc plus *o*eOct4, ^$^vs. pre-MI, ^&^vs. 1d post-MI. (**B**–**E**): Pre-MI, *n* = 20 per group. At 1d post-MI, PBS injection, *n* = 19; cell therapy: vehicle-treated cMSCs, *n* = 20; *oe*c-Myc-treated cMSCs, *n* = 20; *oe*c-Myc plus *oe*Oct4-treated PBMSCs, *n* = 20; *oe*c-Myc plus *si*Oct4-treated PBMSCs, *n* = 20; cMSCs treated with *si*c-Myc alone, *n* = 20; cMSCs treated with *si*c-Myc plus *oe*Oct4, *n* = 19, cMSCs treated with *si*c-Myc plus *si*Oct4, n = 20. At 30 d post-MI, PBS injection, *n* = 10; cell therapy: vehicle-treated cMSCs, *n* = 13; *oe*c-Myc-treated cMSCs, *n* = 14; *oe*c-Myc plus *oe*Oct4-treated PBMSCs, *n* = 19; *oe*c-Myc plus *si*Oct4-treated PBMSCs, *n* = 13; cMSCs treated with *si*c-Myc alone, *n* = 13; cMSCs treated with *si*c-Myc plus *oe*Oct4, *n* = 14, cMSCs treated with *si*c-Myc plus *si*Oct4, n = 11. **F** *n* = 5, each group

To further assess the roles of Oct4-c-Myc in mediating the response of cMSCs to local signals in the injured heart, we injected cMSCs pre-treated with lentiviral vectors encoding Oct4 (*oe*Oct4), Oct4 siRNA (*si*Oct4), vehicle, or in combination with c-Myc siRNA (*si*c-Myc) or c-Myc overexpression (*oe*c-Myc) were randomly transplanted into the hearts of recipient rats subjected to acute MI. Rats with MI were also randomly assigned to receive PBS injection and served as a control group. The 160 animals were randomly divided into eight groups and underwent serial echocardiography studies. Thereafter, all animals were followed-up for 30 days, during which 36 rats died. The surviving 124 rats were then sacrificed and subjected to pathology and molecular biology tests. We evaluated cardiac function and remodeling by echocardiography at 1 and 30 days after cell transplantation. The rationale for a 30-day follow-up was to determine myocardial repair of transplanted cMSCs. After 30 days, Kaplan–Meier survival analysis showed higher survival rate in the rats receiving transplantation of cMSCs transfected with both *oe*c-Myc and *oe*Oct4 than in the rats receiving PBS injection and cMSCs therapy alone (95% in the rats receiving both *oe*c-Myc and *oe*Oct4-treated PBMSCs *versus* 50% in the rats receiving PBS, *p* = 0.016; versus 55% in the rats receiving cMSCs alone, *p* = 0.020), and this effect was canceled in the rats receiving *si*Oct4-treated cMSCs. However, the inhibition of Oct4 was not rescued by c-Myc overexpression. No significant differences were found between the animals receiving cMSCs alone and those receiving cMSCs pre-treated with *oe*c-Myc plus *si*Oct4 or *si*c-Myc plus *oe*Oct4 (Fig. 6A). Echocardiographic studies showed that on day 1 post-infarct, animals in all eight groups developed typical changes of acute heart failure and LV early remodeling, in comparison with data obtained at the baseline levels. These changes included decreased cardiac function index LVFS, dilated LVEDD and LVEDV, and thinning of LVAWd. Significantly, cMSCs transfected with *oe*c-Myc and *oe*Oct4 were more effective than vector-treated cMSCs in the improvement of cardiac function, and the prevention of adverse LV dilatation after MI (Fig. 6B-E). Although transplantation of vector-treated cMSCs revealed slight improvement of LVFS, LVEDD, LVEDV, and LVAWd at 30d post-MI, *oe*c-Myc-treated cMSCs were more effective than PBS in the improvement of these indexes. Moreover, compared with vehicle-treated cMSCs and *oe*c-Myc-treated cMSCs, cMSCs treated with both *oe*c-Myc and *oe*Oct4 further increased the change in LVFS by 3.6- and 2.2-fold, respectively (Fig. 6B), and LVAWd by 75% and 31%, respectively (Fig. 6E), and decreased the changes in LVEDD by 94% and 44%, respectively (Fig. 6C), and LVEDV by 1.0- and 0.5-fold, respectively (Fig. 6D). These beneficial effects were eliminated in the rats receiving *si*c-Myc-treated cMSCs. The indices in the rats receiving PBS indicated sustained exacerbation with decrease in LVEF, dilation of LVEDD and LVEDV, and thinning of LVAWd. Similarly, TTC staining demonstrated the smallest amount of infarct size in the rats receiving cMSCs co-treated with *oe*c-Myc and *oe*Oct4 (Fig. 6F). However, deficiency of c-Myc or Oct4 canceled this decrease. Thus, overexpression of Oct4 in c-Myc-transfected cMSCs enhanced their ability to improve cardiac remodeling after MI.

**Fig. 7:**
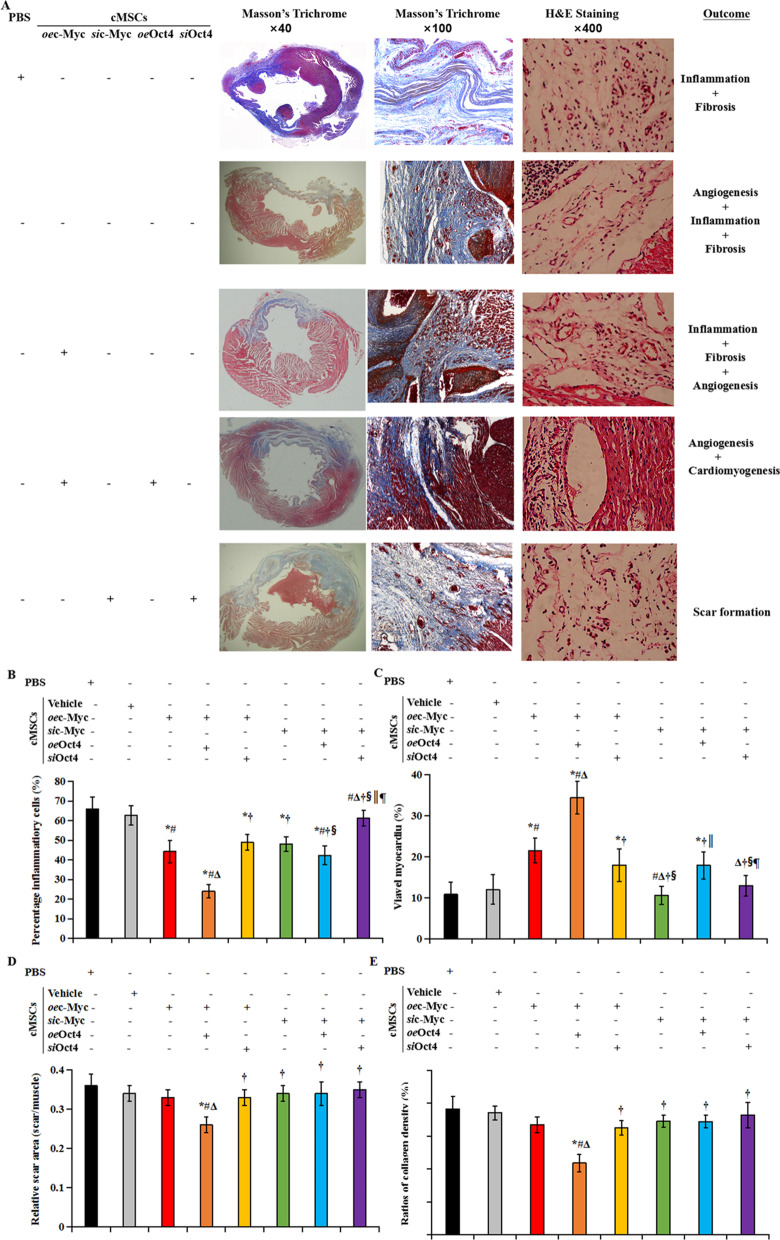
Oct4 collaborated with c-Myc-transfected cMSCs to ameliorate MI pathology. **A** To determine the effects of Oct4 and c-Myc-treated cardiac MSCs on left ventricle structure 30 days after MI, hearts were stained with Masson’s Trichrome (**Left** and **Middle**) or hematoxylin and eosin (**Right**). The infarct zone of the *oe*c-Myc-transfected cMSCs-treated group contained inflammation, fibrosis, and angiogenesis, which were favorably replaced by angiogenesis and cardiomyogenesis after co-transfection of Oct4. However, hearts of rats treated with cMSCs co-transfected with siRNAs of c-Myc and Oct4 displayed extensive fibrosis in the infarct zone demonstrated by collagen formation (blue).The PBS-treated group showed mostly inflammation and scar formation. **B** through **E**. Morphometric measurements of inflammatory cells **B**, viable myocardium **C**, relative scar area **D**, and collagen content (**E**) 30 days after cell therapy revealed a smaller and thicker scar in the *oe*Oct4 plus c-Myc-transfected cMSCs-treated group, compared with alone cMSCs- and PBS-treated groups. Quantitative analysis of viable myocardium **C** in rat hearts from the different groups showed significantly larger ratios of viable myocardium in hearts treated with cMSCs receiving co-transfection of c-Myc and Oct4, compared with alone cMSCs- and PBS-treated groups. *P* value for the difference between groups was calculated by the Welch test followed by a Brown-Forsythe multiple comparisons test. *p* < 0.05: ^*^vs PBS injection*,* ^#^*vs*. cMSCs therapy alone, ^Δ^*vs*. Transplantation of *oe*c-Myc transfected cMSCs, ^†^*vs*. Transplantation of cMSCs transfected with *oe*c-Myc plus *oe*Oct4, ^§^*vs*. Transplantation of cMSCs transfected with *oe*c-Myc plus *si*Oct4, ^║^vs. Transplantation of cMSCs transfected with *si*c-Myc, ^¶^vs. Transplantation of cMSCs transfected with *si*c-Myc plus *o*eOct4 (*n* = 5, each group). cMSCs indicate cardiac mesenchymal stem cells; H&E, hematoxylin and eosin; PBS, phosphate-buffered solution

Postmortem morphometry revealed that compared with PBS injection, cMSCs therapy did not significantly reduce MI-induced inflammation, including neutrophil infiltration, myocyte loss, and bleeding. Transplantation of cMSCs co-transfected with Oct4 and c-Myc caused the greatest reduction of myocardial inflammation, accompanying the greatest increase in viable cardiomyocytes. However, this amelioration was abolished by transfection of Oct4 siRNA (Fig. 7A-C). By microscopic examination, infarcted hearts treated with *oe*c-Myc-transfected cMSCs displayed inflammation, fibrosis, and angiogenesis at the infarct site, and co-transfection of the two siRNAs of c-Myc and Oct4 showed extensive fibrosis with a thicker, fibrotic scar 30 days after MI (Fig. 7A, D, E). These findings probably reflect an anti-fibrotic response of Oct4 in collaboration with the implanted cardiac MSCs. It is interesting to note that a microscopic evaluation revealed cardiomyogenesis in the rats receiving transplantation of cMSCs co-transfected with c-Myc and Oct4. This cardiomyogenesis was mainly located around the blood vessels (Fig. 7A).

**Fig. 8:**
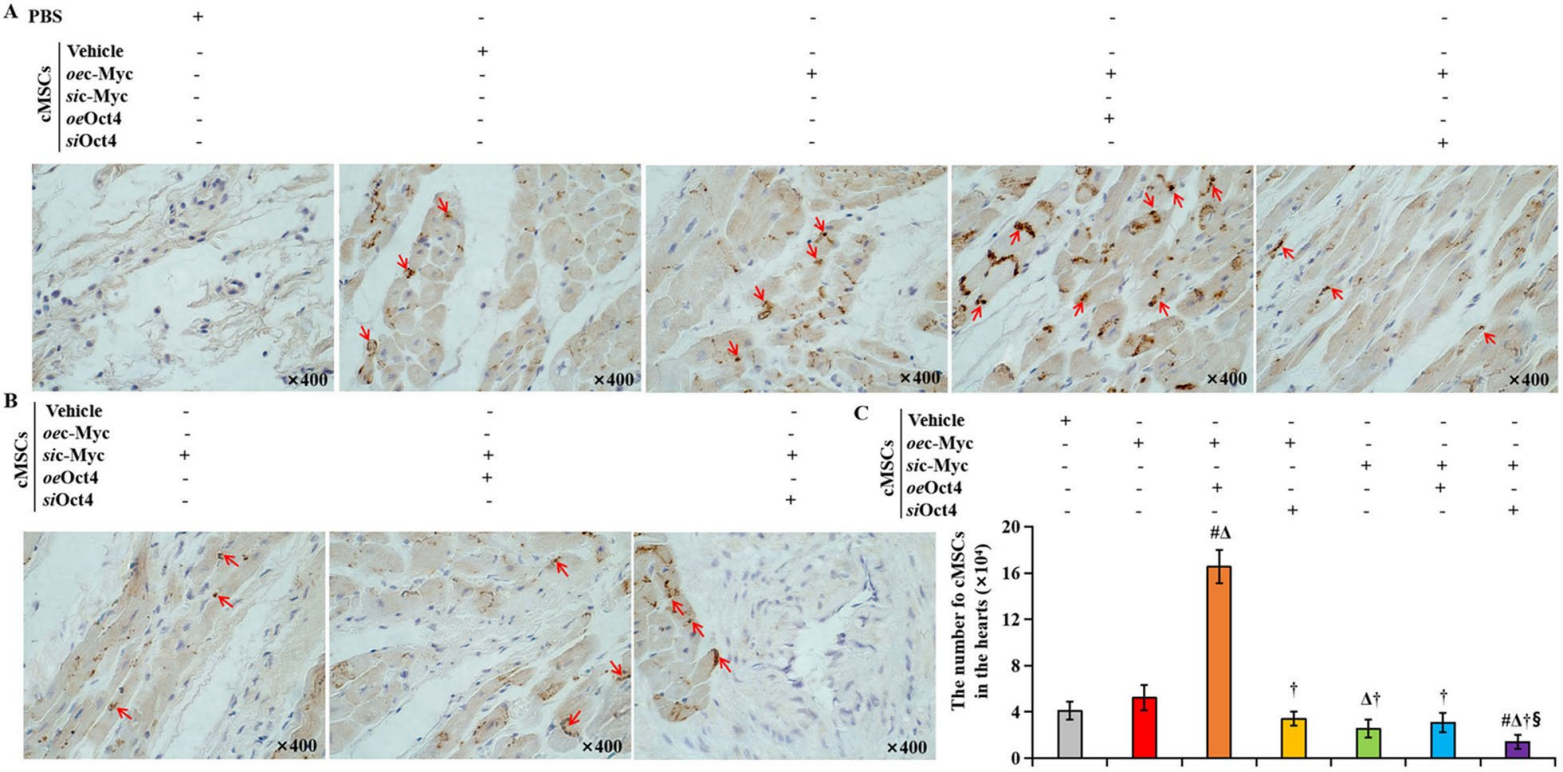

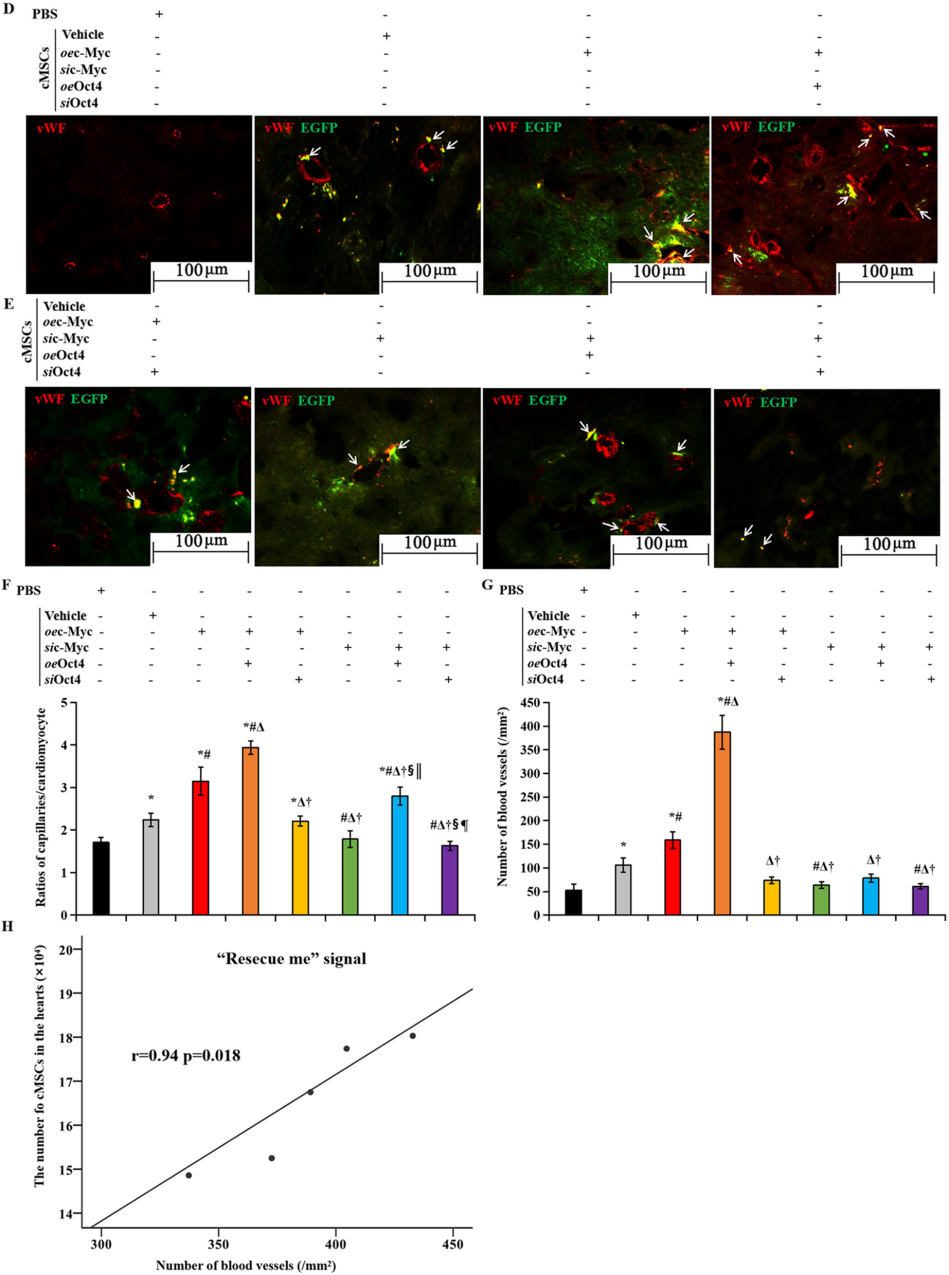
Outcome of the implanted cMSCs. **A** and **B**, Donor cMSCs in the infarcted hearts 30 days after transplantation. To determine the survival of implanted cMSCs (male) in infarcted myocardium (female), the hearts were stained against SRY 30 days after transplantation. Representative microscopic images of the infarct zone: brown nuclear staining indicates transplanted, SRY-positive cMSCs, and blue nuclear staining indicates host cells. cMSCs were found at the residual viable myocardium and were abundant in the heart sections receiving cMSCs co-transfected with *oe*c-Myc and *oe*Oct4 (**A** red arrows). **C** The number of SRY^+^cMSCs presented in the infarcted hearts after 30 days of transplantation. **D** and **E** Double immunostaining with EGFP (green) and anti-vWF (vascular endothelial cytoplasm were stained red) showed that EGFP^+^ cells were gathered around blood vessels and were abundant in the heart sections treated with *oe*c-Myc plus *oe*Oct4-transfected cardiac MSCs. Some of the transplanted EGFP^+^ cells were also stained with vWF (white arrows). **F** Number of capillaries measured by vWF positive capillaries per laminin-outlined cardiomyocyte in transverse heart Sects. 30 days after PBS injection or cell therapy. *P* value for the difference between groups was evaluated using Student’s *t* test. **G** Quantitative data of number of blood vessels in the infarcted hearts 30 days after PBS injection or cell therapy. **C** and **G**: *P* value for the difference between groups was calculated by the Welch test followed by a Brown-Forsythe multiple comparisons test. *p* < 0.05: ^*^vs PBS injection*,* ^#^*vs*. cMSCs therapy alone, ^Δ^*vs*. Transplantation of *oe*c-Myc transfected cMSCs, ^†^*vs*. Transplantation of cMSCs transfected with *oe*c-Myc plus *oe*Oct4, ^§^*vs*. Transplantation of cMSCs transfected with *oe*c-Myc plus *si*Oct4, ^║^vs. Transplantation of cMSCs transfected with *si*c-Myc, ^¶^vs. Transplantation of cMSCs transfected with *si*c-Myc plus *o*eOct4 (n = 5, each group). **H** The correlation between the number of transplanted cells and the number of blood vessels in the *oe*c-Myc + *oe*Oct4 group. cMSCs indicate cardiac mesenchymal stem cells; SRY, sex-determining region Y; EGFP, enhanced green fluorescent protein; PBS, phosphate-buffered solution

All cells were transplanted from male donors into female recipient hearts. Thus, cell retention was assessed by staining for the sex-determining region Y chromosome (Fig. 8A–C). Indeed, we found significant amounts of donor cells at the site of transplantation of cMSCs transfected with *oe*c-Myc plus *oe*Oct4. In contrast, we identified only a few donor cells in the hearts receiving *si*c-Myc/*si*Oct4-treated cMSCs (Fig. 8A, B). Quantitative statistical analysis displayed that the number of cMSCs was greater in hearts treated with cMSCs co-transfected with c-Myc and Oct4 than in the hearts treated with cMSCs alone, or cMSCs transfected with *si*c-Myc or/ plus *si*Oct4, 30 days after MI (Fig. 8C). Moreover, in hearts treated with cMSCs co-transfected with c-Myc and Oct4, a greater increase in the number of engrafted cells was accompanied by a significant compensatory angiogenic response. As shown in Fig. 8F, the capillary-to-myocyte ratio was 1.3-fold greater in the rats treated with cMSCs co-transfected with c-Myc and Oct4, as compared to the rats receiving PBS injection. At the same time, significantly increased values of capillary-to-myocyte ratio were detected in the hearts treated with cMSCs transfected with *oe*c-Myc only or *si*c-Myc plus *oe*Oct4, while a significant reduction in myocardial capillarity was caused by depletion of c-Myc or Oct4. Oct4 also promoted proangiogenic effects of c-Myc in the transplanted cMSCs. To determine the distribution of these transplanted cells in the infarcted heart, we pre-labeled cMSCs genetically with EGFP before transplantation. Notably, EGFP^+^ (plasma stained green) cells were not dispersed throughout the left ventricle but were assembled around blood vessels as revealed by double staining these cells with anti-vWF monoclonal antibodies (Fig. 8D, E). vWF is a marker of blood vascular endothelial cells and communicates angiogenesis. The number of vWF^+^ (plasma stained red) endothelial cells per square millimeter was counted under fluorescence microscope in the peri-infarct regions of hearts from these different treatment groups. The vessel density was increased in *oe*c-Myc-treated cMSCs compared with PBS-treated cMSCs and was even higher in the *oe*c-Myc + *oe*Oct4 treated cMSCs than in the *oe*c-Myc-transfected cMSCs (Fig. 8D, E, G). Moreover, some EGFP-labeled cMSCs expressed vWF (Fig. 8D, E, arrowhead). These cells seemed to switch into a vascular endothelial phenotype. Overexpression of Oct4 promoted the expression of vWF on *oe*c-Myc-transfected cMSCs (Fig. 8D). In addition, the density of transplanted cells was positively correlative with the number of blood vessels assessed by immunofluorescence (Fig. 8G, *r* = 0.94, *p* = 0.018), probably communicating proangiogenic “rescue me” signals to save cMSCs. Together our findings indicate that Oct4 overexpression cooperated with c-Myc to promote MEndoT of implanted cMSCs in vivo, improve their survival, and enhance cardiomyogenesis, which subsequently led to decreased scar formation and inhibition of LV remodeling.

## Discussion

The variations in phenotype and differentiation potential of MSCs in ischemic hearts have been demonstrated to have distinct roles in myocardial repair, some beneficial and some detrimental [31]. In the present study, compared with cMSCs from Sham hearts, cMSCs from Isch hearts showed proinflammatory phenotype with lower anti-inflammation, decreased proangiogenic phenotype, and worse fibrosis. This finding is important because proinflammatory MSCs can aggravate inflammation and myocardial damage [4, 46]. Moreover, cMSCs transplantation alone did not attenuate inflammatory, scarring, and fibrosis compared to PBS injection. However, cMSCs-specific overexpression of Oct4 significantly improved their survival and proliferation consistent with switching proinflammatory cMSCs toward a proangiogenic phenotype. Transplantation of these remodeled cMSCs significantly improved survival, cardiac function and remodeling of rats with MI. Our findings could be of important clinical applications of MSC-base therapy and help provide a strategy to improve the therapeutic effect of transplanted stem cells. To the best of our knowledge, our study is the first to show the potential for negative interaction between cardiac MSCs and the inflammatory or infarcted heart and the key role of Oct4 in cMSCs differentiation away from a proinflammatory to a proangiogenic type in the setting of myocardial ischemia.

The inflammatory microenvironment of AMI has an inhibitory effect on the stem cell potential for regenerating the injured myocardium. Secretion of proinflammatory cytokines hinders their proliferation and favors differentiation [47]. In our study, cMSCs from ischemic hearts secreted proinflammatory cytokines including IL-1*α*, IL-6, MMP2, MMP9, TGF-*β*1, and TNF*α*, as well as inhibited secretion of proangiogenic cytokines including Ang1, bFGF, HGF, VEGF, Tie2, and VEGFR, and anti-inflammatory factor IL-4, causing decrease in their proliferation and angiogenesis, and exacerbation of inflammatory transformation. These unfavorable outcomes of MSCs in ischemic hearts may explain the underlying pathogenetic mechanisms of reported adverse effects in clinical trials. Our findings could be significant for the identification of new targets to improve the use of MSCs therapy by facilitating cell engraftment and functional phenotype transformation. Thus, with smaller proliferation and angiogenesis of cMSCs in ischemic condition, we focused on improving their favorable effects.

Preliminary studies have illuminated that bone marrow MSCs could be reprogrammed into pluripotency by transduction with stemness factors Oct4, Sox2, Klf4, and c-Myc, leading to effective repair of the infarcted heart [48]. Oct4 plays a significant role in the survival and function of various MSCs. Our previous study showed Oct4 as an essential aspect for survival and neovascularization of MSCs in the ischemic conditions [11]. Oct4 can act as a mediator to cooperate with HIF-2*α* to promote proliferation and function of coronary arterial MSCs in ischemic hearts [10]. Furthermore, Oct4 is involved in maintaining and regaining stem cell pluripotency by serving as an exogenous stemness factor [49]. However, few current studies have examined the specific effects of endogenous Oct4 on phenotypic transitions and proliferative ability of cMSCs. In this study, we found that compared with sham cMSCs, Oct4 is downregulated in ischemic cMSCs and consistent with decreased secretion of proangiogenic and anti-inflammatory factors. We explored the consequences of Oct4 upregulation. Oct4 promoted cMSCs proliferation and growth in vitro. Oct4 also promoted expression of angiogenesis-related signaling and tube-forming ability of cMSCs in a hypoxic culture. Our results are in line with the new paradigm of MSC phenotypic transitions [31, 50, 51]. Giallongo et al. [51] showed that MSCs in the inflammatory microenvironment can be induced into a proinflammatory phenotype or a proangiogenic phenotype responding to various stimulation, resulting in different proangiogenic modulatory effects and secretomes in vitro [50]. In the present study, we found that myocardial ischemia switched cMSCs toward an inflammatory phenotype, while cMSCs by overexpression of Oct4 developed a proangiogenic profile. Together, these data support our conclusion that Oct4 has pleiotropic effects on the survival, proliferation, paracrine, and differentiation of cMSCs. Therefore, we conclude that Oct4 has proangiogenic activity and promotes cMSCs angiogenesis.

### The role of Oct4 in cooperation with c-Myc in MEndoT of cMSCs

In the current study, we defined translocation of c-Myc induced by Oct4 as a novel mechanism for MEndoT of cMSCs in hypoxic or ischemic conditions. c-Myc is essential for vasculogenesis and angiogenesis during development and tumor progression [52]. However, the underlying mechanism and the effects of the c-Myc pathway on MEnodT of cMSCs remain unclear. The present study shows that compared with MSCs from Sham rats, MSCs from Isch rats have higher expression of c-Myc accompanied with increased expression of inflammatory and proangiogenic factors. Moreover, c-Myc overexpression further increased expression of proliferation factor Chd1, proinflammatory cytokines (IL-1α, IL-6, TNFα), fibrotic factors (MMP2, MMP9), and proangiogenic factors (Ang1, bFGF, HGF, IGF1, VEGF, Tie2, and VEGFR), leading to simultaneously promote cell proliferation, inflammation, angiogenesis and fibrosis under hypoxic condition. Transplantation of cMSCs overexpressed c-Myc into ischemic hearts caused co-increase in myocardial inflammation, fibrosis, and angiogenesis, resulting in no markedly favorable effects on myocardial repair. By contrast, knockdown of c-Myc expression by siRNA on cMSCs had decreased secretion of proinflammatory and proangiogenic factors. MEndoT contributes to neovascularization of the injured heart and represents a potential therapeutic target for enhancing cardiac repair [53]. In the present study, the positive effect of c-Myc overexpression on MEnodT of cMSCs was offset by its negative effect on inducing inflammatory and fibroblast responses, thus showing ineffective effects on myocardial repair. This finding is paradoxical because exogenous c-Myc has traditionally been considered beneficial for myocardial repair [54]. Yet, it helps to explain why myocardial ischemia switches MSCs toward an inflammatory phenotype and impairs their reparative properties [31]. It may also explain why some clinical trials did not identify that MSCs transplantation could largely promote the recovery of LV function and myocardial viability after AMI [55, 56].

However, direct comparison of gene overexpression showed that cMSCs-specific overexpression of c-Myc *versus* Oct4 resulted in different genomic signatures consistent with the profoundly different hypoxic phenotypes exhibited by these MSCs (i.e., smaller MEnodT with a larger inflammation and fibrosis in c-Myc-overexpressed cMSCs and larger MEnodT nearly lacking inflammation and fibrosis in Oct4 overexpressed cMSCs). Moreover, Oct4 in synergistic combination with c-Myc can undergo phenotypic transition to fully differentiated blood vascular endothelial cells, namely MEnodT. This combination is a negative force for inflammation and fibrosis. The results are of major importance, because they indicate that these 2 transcription factors act cooperatively in regulating stem cell pluripotency [57], resulting in virtually MEndoT by synergistically altering the phenotypic transition of cMSCs. Further investigation of this in vivo cooperation of c-Myc and Oct4 showed that overexpression of Oct4 in c-Myc-transfected cMSCs enhanced their ability to improve animal survival, cardiac remodeling, and function through promoting angiogenesis, anti-inflammatory, and anti-fibrotic response after MI. Conversely, transfection of Oct4 siRNA canceled these beneficial effects of cMSCs therapy. Moreover, transplantation of cMSCs transfected with Oct4 siRNA increased scar thickness. This finding is important because, on the basis of Laplace’s law, increased scar thickening leads to wall thinning, and subsequent infarct expansion and LV remodeling [31]. Indeed, implanted Oct4 siRNA cMSCs resulted in significant LV dilation and impaired cardiac function after MI.

Further studies on the relationship between Oct4 and c-Myc surprisingly showed that Oct4 overexpression did not significantly affect c-Myc expression but regulate c-Myc translocation. Oct4 overexpression upregulated multiple known c-Myc targets through c-Myc nuclear-to-cytoplasm translocation, leading to enhancement of MEnodT. c-Myc is known to localize in the nucleus and nucleoli [58]. Nuclear c-Myc is readily degraded [59]. In the present study, large levels of c-Myc were translocalized to the cytoplasm in Oct4 overexpressed cMSCs, but we did not detect increased level of total c-Myc. Similarly, Oct4 expression was significantly higher in the cytoplasm than in the nuclei. Conversely, c-Myc mainly gathered surrounding the nuclei of cMSCs after Oct4 siRNA interference. This suggests that Oct4 mediated c-Myc translocation from the nucleus to the cytoplasm, preventing c-Myc degradation. ChIP assay confirmed that the c-Myc binding motif overlapped with the Oct4 binding site. Simultaneously, upregulation of the nucleus-to-cytoplasm translocation of c-Myc was accompanied by coordinated activation of VEGF signaling including VEGF, VEGFR2, MAPK, and AKT after Oct4 overexpression. VEGF signaling plays a key role in the formation and growth of blood vessels. Inspection of VEGF signaling-mediated MEnodT revealed that a neutralizing antibody against VEGF-A can efficiently block endothelial marker expression and inhibit cell angiogenesis [60]. Taken together, Oct4 interacts with c-Myc within the cytoplasm, which activates VEGF signaling, resulting in increased MEnodT and angiogenesis of cMSCs under hypoxic condition (Additional file 5: Fig. S4).

Furthermore, we identified Oct4 and c-Myc cooperated to increase cell retention and angiogenesis in vivo. Based on our in vitro culture proliferation assays and differentiation assays, we postulate that in the context of myocardial ischemia, Oct4 overexpressed cMSCs were assembled around blood vessels and differentiated toward a vascular endothelial phenotype. Consistent with this phenotypic transition, was the promotion of the proangiogenic paracrine action, which enhanced vascular formation.

c-Myc/VEGF signaling axis could mediate this effect because our microarray indicated it is expressed in Oct4 overexpressed cells. These results are consistent with our previous findings showing that VEGF expression was upregulated in Oct4 overexpressed MSCs and promoted angiogenesis and engraftment [10]. Transgenic studies have shown that c-Myc is necessary for VEGF expression in ES cells and in vivo, and is sufficient to induce an angiogenic response [52]. In the current study, Oct4 binds to and cooperates with c-Myc to activate some intracellular signaling pathways, including angiogenesis, cell activation, and anti-inflammatory system process, leading to the activation of cell proliferation and MEndoT, as well as inhibition of inflammation and fibrosis under hypoxic condition. These findings allow us to reinterpret the meaning of Oct4 in cMSCs phenotypic transitions in the context of MI. Indeed, Oct4 also cooperated with c-Myc to promote MEndoT of cMSCs in the ischemic hearts, resulting in further improvement of animal survival and cardiac function, and attenuation of LV remodeling after MI. Decreased Oct4 or c-Myc levels reflected exacerbated myocardial death, inflammatory response, and fibrosis. These factors have detrimental effects on the myocardium by increasing infarct size and attenuating heart function. The strong synergistic effects of Oct4/c-Myc against myocardial injury suggest that those patients with high expression levels of Oct4 and c-Myc in MSCs post-MI may represent a distinct group that will benefit from MSCs treatment.

An unusual finding was the presence of islands of cardiomyogenesis surrounded by blood vessels in the infarcts of rats treated with cMSCs co-transfected with Oct4 and c-Myc. MSCs have the proven capacity to differentiate into blood vascular endothelial cells (ECs) [61] and promote the neovascularization capacity of ECs, which is associated with improved myocardial protection and anti-fibrotic effects on the heart [62]. The synergistic effects of c-Myc and Oct4 on MEndoT capacity of cMSCs could partially explain the unique finding of cardiomyogenesis. It is interesting to note that engrafted cMSCs located around blood vessels and expressed vWF, a marker of blood vascular ECs, which communicates vasculogenesis and activates their rescue properties through an adaptive reparative response on the heart [63]. We have shown that although c-Myc overexpression induced vasculogenesis of cMSCs, it simultaneously caused an inflammatory response, which did not lead to increased survival of transplanted cMSCs. Co-overexpression of Oct4 can be an advantage to avoid an inflammatory response by c-Myc and amplify its angiogenesis effect. It seems that cooperation of c-Myc and Oct4 improves implanted cMSCs survival and their therapeutic effects. The positive synergistic effect between c-Myc and Oct4 in our study gives clinical evidence of the possible utility of c-Myc/Oct4 as a therapeutic target to improve the efficacy of cell therapy.

## Conclusions

Altogether, our work proposes that the synergetic cooperation between c-Myc and Oct4 could promote MEndoT capacity of cMSCs, diminish the negative effects of cardiac inflammation on the MSC’s reparative properties, enhance cell retention, and improve the outcome of cell therapy after MI.

## Supplementary Information

**Additional file 1:** An expanded Materials and Methods section.

**Additional file 2: Fig. S1.** Flowchart of cell preparation, gene transfection, culture, transplantation, echocardiography, and histopathologic evaluation.

**Additional file 3: Fig. S2.** Characterization of MSCs from myocardial ischemic rats.

**Additional file 4 : Fig. S3.** c-Myc and Oct4 contribute differently to the in vitro growth of cMSCs.

**Additional file 5: Fig. S4.** Proposed mechanism of Oct4 overexpression induced cytoplasmic translocation of c-Myc.

**Additional file 6: Table S1.** Primers for qRT-PCR.

**Additional file 7: Table S2.** The antibodies for western blotting, immunofluorescence, and immunohistochemistry, respectively.

**Additional file 8:** WB original pictures in Figure 2F.

**Additional file 9:** WB original pictures in Figure 4E.

**Additional file 10:** WB original pictures in Figure 5I.

## Data Availability

The datasets used and/or analyzed during the current study are available from the corresponding author on reasonable request.

## Abbreviations

AMI: Acute myocardial infarction
Ang-1: Angiopoietin 1
Bfgf: Basic fibroblast growth factor
cMSCs: Cardiac-resident mesenchymal stem cells
FACS: Flow cytometry analysis
IVSd: Interventricular septal thickness in diastole
Isch: Ischemia
LAD: Left anterior descending coronary artery
LV: Left ventricular
LVEDD: Left ventricular end-diastolic dimension
LVESD: Left ventricular end-diastolic dimension
LVEF: Left ventricular ejection fraction
LVFS: Left ventricular fractional shortening
LVEDV: Left ventricular end-diastolic volume
LVESV: Left ventricular end-systolic volume
LVPW: Left ventricular posterior wall thickness
MEndoT: Mesenchymal-to-endothelial transition
MI: Myocardial infarction
MNs: Mononuclear cells
MSCs: Mesenchymal stem cells
PbMSs: Peripheral blood MSCs
TTC: Triphenyltetrazolium chloride
Tx: Cell therapy
VEGF: Vascular endothelial growth factor
