# Supplementary material for: Oct4 cooperates with c-Myc to improve mesenchymal-to-endothelial transition and myocardial repair of cardiac-resident mesenchymal stem cells
Source: Stem Cell Res Ther. 2022 Sep 2;13:445. doi: 10.1186/s13287-022-03120-7 (PMC9438134; doi:10.1186/s13287-022-03120-7)
Supplement: Supplementary file 1 — Additional file 1: An expanded Materials and Methods section. [file 13287_2022_3120_MOESM1_ESM.doc]

**Research Design and Methods**

**Antibodies and Other Reagents**

Transwell six-well plates were obtained from COSTAR The ABsolute QPCR SYBR Green premix was purchased from Takara. Supplementary **Tab. S1** presents the sequences of primers used in this study. Supplementary **Tab. S2** lists the details of the antibodies. 4′,6-diamidino-2-phenylindole (DAPI; catalog 28718-90-3) was purchased from Sigma-Aldrich.

**Animals**

Inbred Lewis rats (18 months old) were used. The Animal Care and Use Committee of GuangZhou Red Cross Hospital Medical College of Ji-Nan University approved all the animal experiments, which were conducted in compliance with the Guide for the Care and Use of Laboratory Animals published by the National Academy Press. Rats were euthanized by CO2 inhalation upon completion of the study.

**Rat Models and Experimental Protocol**

The model of myocardial ischemia (Isch) and myocardial infarct (MI) in the rats has been previously described ^[1, 2, 3]^. In brief, rats were weighed and anesthetized with a mixture of ketamine/xylazine (100/15 mg/kg, IP), and the chest was opened by a left thoracotoy. A 7-0 prolene suture was used to ligate the left anterior descending coronary artery at the lower border of the left atrium. MI was confirmed by ST segment elevation in the electrocardiogram, presence of regional cyanosis in the myocardium, and left ventricular regional wall motion hypokinesia on echocardiography. Animals with an ejection fraction (EF)<70% and fractional shortening (FS)<35% evaluated by echocardiography after induction of MI were selected ^[1]^. For sham Isch, the suture was passed around the coronary artery and removed without ligation.

**Isolation, Expansion, and Purification of MSCs**

To evaluate the effects of myocardial ischemia (Isch) on resident cardiac MSCs (cMSCs) and peripheral blood MSCs (pbMSCs), we isolated MSCs from the heart and the abdominal aortic blood of male Lewis rats with Isch 30 days after MI (n=10) or sham MI (n=10), and cultured via the adherent culture method, as described previously ^[4, 5]^. Briefly, cells were extracted with an enzymatic digestion mixture using 3 cycles of incubation at 37°C for 10 minutes. Plastic-adhered cells were incubated at 37°C in humid air with 5% CO_2_ and grown in DMEM (Biological Industries) with 15% FBS (Biological Industries), 1% penicillin-streptomycin (Biological Industries), 1% L-glutathione (Sigma-Aldrich), 1% MEM nonessential amino acids (Invitrogen), and 0.1 mM 2-mercaptoethanol (Invitrogen). The medium was changed 3 days after plating and subsequently every 3 or 4 days. The fourth generation cells were used for subsequent experiments including their purity, viability, characteristics, pluripotency, gene transfection, and transplantation, as shown in **Fig. S1**.

***In vitro* Directed Differentiation of MSCs**

The MSCs underwent direct differetiation toward osteogenesis, chondrogenesis, adipogenesis, and angiogenesis by growth factor supplementation and growth on defined matrices. For osteogenesis, the MSCs were induced with a osteogenic differentiation medium kit (HUXUB-90021, Cyagen, Soochow, China) and cultured for 21 days, alizarin red staining was performed to evaluate osteogenic products as previously described ^[6]^. For chondrogenesis, the PBMSCs were induced for 21 days using a chondrogenic differentiation medium kit (HUXUB-9004, Cyagen, Soochow, China) for 14 days and evaluated by alcian blue staining for differentiation identification ^[7]^. For adipogenesis, the PBMSCs were cultured for 21 days by a adipogenic differentiation medium kit (HUXUB-9004,Cyagen, Soochow, China). The formation of lipid vacuoles was assessed by Oil Red O staining ^[8]^. For vascular differentiation, growth factors bFGF (5 ng/ml; Invitrogen), and VEGF (20 ng/ml; R&D Systems; Minneapolis, MN, USA) were added. The capillary-like structure was viewed 6 h later. Microscopic fields containing the tube structure that formed on the gel were photographed using fluorescence inverted phase contrast microscopy. Five fields per test condition were examined ^[9]^. Angiogenesis was detected using immunofluroscence withfactor VIII and alpha smooth muscle actin (α-SMA) double positive- staining.

**Hypoxic or normoxic treatment**

Cells were removed and exposed to hypoxic (37 ℃, 93% N_2_, 5% CO_2_, and 2% O_2_) oxygen levels in a water-jacketed CO_2_ incubator; the hypoxic oxygen level was maintained via regulated nitrogen injection (Forma Scientific). Cells cultured under standard conditions (21% O_2_, 5% CO_2_, and 74% N_2_) served as normoxic control cultures. The hypoxic condition was maintained throughout the performance of all subsequent analyses.

**Fluorescence Activating Cell Sorter (FACS)**

To define the phenotype of cMSCs and pbMSCs, cultured cells at passage 4 were immunostained in FACS buffer (0.1% BSA in PBS) with the following MSC surface marker antibodies: SH2, SH3, CD90, CD147, CD34, CD45 and CD133. Mouse IgG1, IgG2a, and IgG2b (Becton Dickinson) were used as isotype controls, and marker expression was evaluated using FACS. To evaluate the effects of c-Myc and Oct4 on the proliferation of cMSCs in hypoxic condition, we cultured ischemic cMSCs transfected with a lentivirus encoding overexpressed c-Myc (*oe*c-Myc), Oct4 (*oe*Oct4), or vehicle (served as control, CON) for 48 hours under hypoxic or normoxic conditions. Cell proliferation was assessed using fluorescence staining and FACS for proliferation marker (5-bromodeoxyuridine, BrdU) following the company's protocol.

**Cell Viability and Proliferation**

For the cell viability assay, MSCs were seeded at 2 × 10^3^ cells/well on a 96-well plate. Cell viability was assessed by visual cell counts after performing a trypan blue exclusion assay. Cell growth was measured by a cell counting kit-8 (CCK-8, Sigma) according to the manufacturer’s protocol in the diferent time of 0, 24 h, 48 h, 72 h, and 96 h after diferent treatments. Briefly, after treatment, the CCK-8 solution was added to the culture medium and incubated at 37°C for 48 h. The absorbance was read at 450 nm with a microplate reader (Bio-Rad, Hercules, CA, USA) ^[10]^. Cell viability was calculated by experimental group absorbance value/control group absorbance value. Cell proliferation was assessed using fluorescence staining and FACS for proliferation markers (BrdU and Ki67) following the company's protocol. For BrdU incorporation studies, BrdU labeling reagent (Amersham) was diluted 1:1000 in medium to a final concentration of 10 μM BrdU, and sterilized through a 0.22 μm filter. 2 ml of staining solution were added to each well of a 6-well plate, followed by incubation for 12, 24, 36, or 48 hours as indicated. After washing, fixation, and incubation, 20 μl anti-BrdU antibody (Becton-Dickinson) was added, followed by 30 minute incubation at room temperature in the dark. Flow cytometry was done on a FACScan (Becton-Dickinson) and results were analyzed using FlowJo software (TreeStar) ^[11]^. Ki-67 immunofluorescence was performed on coverslips in 12-well-plates and after 24-h incubation in 37 °C CO2 incubator; the cells were rinsed with 1 × PBS and fixed in 4% formaldehyde for 15 min. Cell membrane was permeabilized with 0.1% triton X100 diluted in PBS and for minimizing the risk of unspecific binding, cells were treated with blocking buffer containing 5% horse serum diluted in PBS for 1 h. Then cells on coverslip were incubated overnight with primary antibody against Ki-67 at 4 °C overnight. Then cells were rinsed in PBS 3 × 5 min and incubated with the secondary antibody for 1 h at room temperature in the dark. At last, coverslips were sealed by adding mounting medium containing DAPI. Negative staining controls were incubated with the same TBS buffer without the primary antibody. Photomicrographs were taken at ×20 magnification in four randomly selected fields in each well. Four observers (FCS, MFS, LK and SEB), blinded to clinical data, independently evaluated the whole area of the tumors. Discrepancies were solved by further review by all the observers and a consensus was reached.

**Angiogenesis Array**

To define the levels of angiogenesis-related cytokine secretion from cMSCs and pbMSCs, we used an Angiogenesis Protein Array kit (QAR-ANG-100, RayBiotech) according to the manufacturer's instructions ^[12]^. For all multiplex assays, samples were run in triplicate wells. Expression of genes encoding 60 angiogenesis-related cytokines was evaluated in cMSCs and pbMSCs from ischemic (Isch) or sham operation (Sham) hearts. All data are deposited in the Gene Expression Omnibus (accession number GSE126168). Concentrations of cytokines were determined by Quasys Q-View imaging and a software system. For all multiplex assays, samples were run in triplicate wells.

**Immunofluorescence Staining**

Cells or tissues seeded or fixed on lysine-treated glass coverslips were washed with PBS, fixed with 4% paraformaldehyde (Sigma), permeabilized by 0.5% Triton X-100 for 10 min, and pre-blocked with 5% Bovine Serum Albumin for 1 h. Immunofluorescence staining was performed by incubation with primary antibodies (**Tab. S2**) at 4 °C overnight, followed by secondary antibodies Alexa-488 anti-mouse (A32723;1:200;Thermo Fisher Scientific, Inc.) or Alexa-594 anti-rabbit (A32732;1:200; Thermo Fisher Scientific, Inc., Waltham, MA, USA) for 1 h at room temperature. Cells were washed extensively with 0.1% tween-20 in PBS and mounted with 4’,6-Diamidino-2-phenylindole dihydrochloride (DAPI, S33025; Thermo Fisher Scientific, Inc.). Fluorescent signal was captured by Olympus FV1000 confocal microscope (Olympus) and analyzed by FV10-ASW software (Olympus).

**Gene Expression Heatmaps**

Gene expression heatmaps were performed by Miltenyi Biotec Genomic Services as previously described ^[13]^. Briefly, total RNA was isolated and used to synthesize double-stranded cDNA, and then biotinylated antisense complimentary RNA (cRNA) was generated through transcription using a Genechip Expression 3'-Amplification Reagent for IVT Labeling kit (Affymetrix, Inc. USA). Next, the biotinylated labeled cRNA was hybridized to the Affymetrix Rat 230 2.0 GeneChip array (P/N900470, Affymetrix Inc., USA), stained with an antistreptavidin antibody, followed by a second staining step using a streptavidin-phycoerythrin conjugate. Fluorescence was detected using the Genechip System Confocal Scanner (Hewlett-Packard), and analysis of the data from each GeneChip was conducted using the GeneChip 3.1 software produced by Affymetrix, using the default settings. Heatmaps were generated using the R add-on package pheatmat to reflect gene expression values under several conditions ^[14]^.

**Quantitative real-time reverse transcriptase polymerase chain reaction (qRT-PCR)**

Messenger RNAs from cultured MSCs were isolated using commercial kits. Total RNA was extracted using RNA-Stat (Iso-Tex Diagnostics, Friendswood, TX, USA) according to the manufacturer’s instructions. The extracted RNA (500 ng) was converted into cDNA using Taqman Reverse Transcription Reagents (Applied Biosystems, Foster City, CA, USA). All probes and primers were designed using Express Primer 3 software developed by the Whitehead Institute for Biomedical Research (Cambridge, MA, USA). The nucleotide sequences of selected genes were obtained from GenBank, and the primer information is shown in **Tab. S1**. The increase in fluorescence of 6-carboxyfluorescein (6-FAM) was automatically measured during quantitative real-time polymerase chain reaction (qPCR). Cycle thresholds (CT) for the individual reactions were determined using the ABI Prism SDS 2.0 data processing software (Applied Biosystems). Relative mRNA transcript levels were quantified using the 2^-ΔΔ^ CT method, with GAPDH as an internal control.

**Western blotting**

To confirm the levels of proteins of interest, western blotting assays were performed as described previously, with modifications ^[12]^. In brief, protein extracts (100 μg per sample) were separated using sodium dodecyl sulfate polyacrylamide gel electrophoresis (SDS-PAGE; Bio-Rad Laboratories, Hercules, CA, USA) and electrotransferred onto polyvinylidene difluoride (PVDF) membranes (GE Healthcare, Piscataway, NJ, USA). The membranes were probed with primary antibodies (**Tab. S2**) and then with labeled secondary antibodies. GAPDH and β-actin served as a positive control. The target protein levels were determined as the ratios of the target protein/GAPDH using Image-Quant software (GE Healthcare).

**β-Catenin and Oct4 Transfection**

Retroviral plasmid vectors, pMXs, expressing β-catenin or Oct4, were transfected with pReceiver-LV233 lentiviral vector (GeneCopoeia (Rockville, MD) into cMSCs with the Fugene HD reagent, as directed by the manufacturer’s instructions. A pSi-LVRU6GP vector with a puromycin resistance cassette (GeneCopoeia, Rockville, MD, USA) was used to express small interfering RNAs (*si*RNAs) to knock down c-Myc & Oct4 expression. c-Myc (*oe*c-Myc) or Oct4 (*oe*Oct4) overexpression and c-Myc (*si*c-Myc) or Oct4 (*si*Oct4) deficiency were induced by transfecting the cells with vectors encoding c-Myc or Oct4, c-Myc siRNA, or Oct4 siRNA, respectively. Control siRNA duplexes were used as the control (CON).

**Bulk RNA-seq Analysis**

Total RNA was isolated using Trizol (Invitrogen) from cMSCs isolated from Isch or Sham hearts, or cMSCs overexpressed with or without c-Myc or Oct4, and cultured for 48 h under hypoxic conditions. The RNA library were pprepared as per the HiSeq 2500 RNA-seq platform (Illumina, San Diego, CA, USA). RNA-seq data were counted over gene exons using featureCounts 2.0.1. Genes were annotated according to the Rattus_norvegicus.Rnor_6.0.104 annotation file ^[15]^. The DESeq2 Bioconductor R package was used to identify differentially expressed genes at a 5% false discovery rate (P value adjusted≤0.05) by using the Benjamini-Hochberg procedure to adjust P values ^[16]^. We used Ingenuity Pathway Analysis to perform Gene set enrichment analysis. Significantly enriched pathways were identified using a 5% false discovery rate cutoff, and their enrichment significance was quantified using −log_10_ of P value adjusted ^[17]^. Data are expressed as pathways downregulated or upregulated in overexpressed c-Myc or Oct4 cMSCs compared with control cMSCs. The raw counts were loaded into R 4.1.0 (R Foundation for Statistical Computing, Vienna, Austria) for statistical analysis. We used the pheatmap function to perform hierarchical clustering analyses.

**Transcriptome Analysis**

To analyze gene expression upon ischemia, total RNA were isolated from the Isch or Sham cMSCs by using Trizol, purified using an RNeasy Micro Kit, and sequenced using Illumina HiSeq 2000. Alignment was performed on 100bp paired-end samples using Tophat 2.0.6. Reads were aligned to hg19. The Defined Region Differential Seq program was proceeded to evaluate differential expression P-values. We analyzed the genes with differential expression (FDR<0.05) between the Sham c-MSCs and the Isch c-MSCs using GO-Elite (http://www.genmapp.org/go_elite/) ^[18]^. We used Whole Genome rVISTA ^[19]^ to identify enriched predicted transcript factor (TF) binding sites among these gene sets.

**Tube Formation Assay**

MSCs were seeded on matrigel coated 96-well plates, and transfected with either Oct4 or a control vector. After 72 h of hypoxic culture, we observed the tube formations of MSCs in the liquid matrigel (BD Biosciences, USA, BD Matrigel Matrix Cat. No. 356234) according to the manufacturer's instructions. Briefly, the wells were coated with 10 μL of Matrigel and incubated at 37°C for 30 minutes to allow gelation. Then, 1.5 × 10^4^ cells/well were suspended in EGM‐2 supplemented with 2% FBS and plated onto a layer of Matrigel. The angiogenesis plate was then incubated for an additional 72 hours at 37°C under hypoxic conditions, and capillary‐like tube formation was observed and photographed with a microscope. Each well was digitally photographed under a phase contrast microscope (Leica) . The observed tubes were counted ^[20]^. Four representative fields are counted and the average of the total area of complete tubes formed by cells per unit area is compared by Image-Pro Plus®.

**Chromatin immunoprecipitation assays (CHIP)**

About 2.0 × 10^6^ cells were used in each ChIP experiment. The proteins were cross-linked by incubating cells with 1% formaldehyde for 10 min. Nuclear extraction and chromatin digestion was performed. Sheared chromatin was diluted and immunoprecipitated with 2 μg of an anti-c-Myc or control IgG antibody, DNA-protein complexes were eluted and purificated subsequently. ChIP assays were performed according to manufacturer's protocol from a ChIP assay kit (Merck Millipore). The DNA samples were detected by using real-time PCR analysis. To amplify the c-Myc binding site in the Oct4 promoter, the primers sequences was as shown in **Tab. S1**.

**EGFP labelling**

At 24 h after transfection with the *oe*c-Myc, *sh*c-Myc, *oe*Oct4, *si*Oct4, or control vectors into male cMSCs, cells were co-transfected with a lentiviral vector containing enhanced GFP (EGFP) cDNA, as described previously ^[21]^. More than 70% of cMSCs were EGFP-positive, as determined by flow cytometry.

**Cell Therapy**

Male rats and female rats were used as donors and recipients, respectively. For cell transplantation experiments, we injected the male cMSCs transfected with *oe*c-Myc, *si*c-Myc, *oe*Oct4, *si*Oct4, or control vectors, or phosphate-buffered saline (PBS, 20 ul) into the ischemic border zone (5×10^6^ cells, four sites, 5 µl per site, 1–2 cm apart) 1 minute after LAD occlusion. Female rats with LVFS≥35% at day 1 after MI were excluded from the study.

**Echocardiography**

Light anesthesia was induced by inhalation of 2% isoflurane/98% O_2_ and subsequently maintained by 0.5% to 1% isoflurane. Rats underwent echocardiography in a 7.5-MHz phased-array transducer (Acuson Sequoia 256, Siemens, Mountain View, CA), and were operated by an experienced technician blinded to treatment group identity; two-dimensional images were obtained at the mid-papillary and apical levels. LV end-diastolic volume, and internal diameter at diastolic phase (LVEDv and LVEDd, respectively) were measured using the biplane area-length method. LVFS was calculated according to the modified Simpson method: FS (%) = [(LVIDd-LVIDs)/LVIDd] × 100, where LVID is LV internal dimension, s is systole, and d is diastole. All measurements were averaged for three consecutive cardiac cycles and performed by an experienced technician who was blinded to the treatment groups.

**Histology**

To evaluate the infarct size 30 days after cell therapy, hearts were harvested and frozen at -80°C. The frozen hearts were cut transversely into 1.2-mm-thick slices and stained with 1% 2,3,5-triphenyltetrazolium chloride (TTC) in PBS (pH 7.4) for 20 minutes in a 37°C water bath. After fixation for 4 to 6 hours in 10% neutral buffered formaldehyde, both sides of each slice were photographed. Viable myocardium stained brick red, and infarct tissues appeared pale white. Infarct and LV area were measured by automated planimetry using Image J software, with the infarct size expressed as a percentage of the total LV area.

Infarcted rat heart samples were fixed by 4% paraformaldehyde (PFA) solution, embedded in paraffin, and cut into 4 µm transverse sections at different levels. Hematoxylin and eosin (H&E) and Masson’s trichrome staining were performed on paraffine-mbedded sections at papillary and apical level and then examined under ordinary polychromatic light or polarized light microscope to determine extent of cardiac inflammation, fibrosis, and cardiomyogenesis. For each section, ten to fifteen images were acquired from randomly selected fields in infarct and non-infarct areas. Images analysis was conducted using Image J software (National Institutes of Health). The severity of inflammation damage was evaluated as the percent of inflammatory cells within peri-infarct regions. Viable myocardium was calculated by multiplying myocardium density by viable myocardial volume. The percent value of viable myocardium area were estimated by image tool 3.0. Infarct size was determined by planimetric measurement with a digital image program (Scion ImageJ) and calculated by dividing the sum of the planimetered endocardial and epicardial circumferences occupied by the infarct by the sum of the total epicardial and endocardial circumferences of the LV on three transversal sections from the apex to the base. Relative scar area was computed as the ratio of nonviable to total pixels in the LV. Collagen density was calculated as the ratio of positive staining area to the total scar area.

**Engraftment and vasculogenesis**

Tissue sections of rat hearts receiving PBS injection or cMSCs therapy were mounted on charged glass slides, deparaffinized, and digested using standard protocol for *in situ* hybridization. Hearts from the female rats receiving male cMSCs injection were used to assess cell engraftment by staining for the sex-determining region Y chromosome. Studies were made by using Y chromosome-specific DNA probe labeled with biotin-conjugated with anti-Digoxin for chromosome. Slides were washed three times with precooled PBS and fixed with 4% paraformaldehyde for 10 min at room temperature. The probe was hybridized with tissues at 42 °C in the dark overnight. The next day, tissue sections were washed five times with saline-sodium citrate buffer at various concentrations, followed by incubation with streptavidin-HRP, and the sections were stained with 3,3’-diaminobenzidine solution and counterstained with hematoxylin solution. Engraftment was evaluated by determining the number of Y chromosome-positive cells at the site of transplantation.

To determine vasculogenesis of cMSCs, immunofluoroscence staining was performed on a series of the cryostat sections. Vascular differentiation was evaluated by calculating the proportion of cells that expressed both EGFP and vWF relative to all EGFP-positive cells using anti-vWF antibody. Vessel density was expressed as the number of vWF^+^ endothelial cells per square millimeter. Alexa Fluor 488-conjugated goat anti-rabbit IgG was also used for visualization of anti-laminin antibody to analyze capillary density (capillaries/cardiomyocyte). Cross-sectional area and numerical density of laminin-outlined transversely cut cardiac myocytes were determined in the same regions used for capillary analysis, and a capillary-to-myocyte ratio was calculated based on the numerical densities calculated for capillaries and cardiac myocytes, as previously detailed ^[22]^. A pathologist who was blinded to group identity evaluated the capillary density and cell count by counting vessels and cells in the chosen areas.

**Statistical analysis**

Data are presented as the mean ± standard error of the mean (SEM). Discrete variables are presented as frequency and proportion. By performing normality test (Shapiro-Wilk test) and homogeneity test of variance, the data that satisfy normal distribution and equal variance assumptions were used for one-way ANOVA analysis of these variables. When the data were conferred for normal distribution but non-homogeneity of variance, Welch ANOVA analyses were performed. Comparisons were performed using the x^2^ or Fisher’s exact test for discrete variables. A 95% confidence interval (CI) (p < 0.05) was considered significant.

**References**

1. Ji Z, Chen S, Cui J, Huang W, Zhang R, Wei J, Zhang S. Oct4-dependent FoxC1 activation improves the survival and neovascularization of mesenchymal stem cells under myocardial ischemia. Stem Cell Res Ther. 2021; 12: 483. doi: 10.1186/s13287-021-02553-w

2. Zhao L, Zhang R, Su F, Dai L, Wang J, Cui J, Huang W, Zhang S. FoxC1-induced vascular niche improves survival and myocardial repair of mesenchymal stem cells in infarcted hearts. Oxid Med Cell Longev. 2020; 2020: 7865395. doi: 10.1155/2020/7865395

3. Zhao L, Zhang S, Cui J, Huang W, Wang J, Su F, Chen N, Gong Q. TERT assists GDF11 to rejuvenate senescent VEGFR2^+^/CD133^+^ cells in elderly patients with myocardial infarction. Lab Invest. 2019; 99: 1661-1688. doi: 10.1038/s41374-019-0290-1

4. Pan M, Wang X, Chen Y, Cao S, Wen J, Wu G, Li Y, Li L, Qian C, Qin Z, Li Z, Tan D, Fan Z, Wu W, Guo J. Tissue engineering with peripheral blood-derived mesenchymal stem cells promotes the regeneration of injured peripheral nerves. Exp Neurol. 2017; 292: 92-101. doi: 10.1016/j.expneurol.2017.03.005

5. Naftali-Shani N, Levin-Kotler LP, Palevski D, Amit U, Kain D, Landa N, Hochhauser E, Leor J. Left ventricular dysfunction switches mesenchymal stromal cells toward an inflammatory phenotype and impairs their reparative properties via toll-like receptor-4. Circulation. 2017; 135: 2271-2287. doi: 10.1161/CIRCULATIONAHA.116.023527

6. Mihaila SM, Frias AM, Pirraco RP, Rada T, Reis RL, Gomes ME, Marques AP. Human adipose tissue-derived SSEA-4 subpopulation multi-differentiation potential towards the endothelial and osteogenic lineages. Tissue Eng Part A. 2013; 19: 235-246. doi: 10.1089/ten.TEA.2012.0092

7. Hu X, Zhu J, Li X, Zhang X, Meng Q, Yuan L, Zhang J, Fu X, Duan X, Chen H, Ao Y. Dextran-coated fluorapatite crystals doped with Yb3^+^/Ho3^+^ for labeling and tracking chondrogenic differentiation of bone marrow mesenchymal stem cells in vitro and in vivo. Biomaterials. 2015; 52: 441-51. doi: 10.1016/j.biomaterials.2015.02.050

8. Martella E, Bellotti C, Dozza B, Perrone S, Donati D, Lucarelli E. Secreted adiponectin as a marker to evaluate in vitro the adipogenic differentiation of human mesenchymal stromal cells. Cytotherapy. 2014; 16: 1476-1485. doi: 10.1016/j.jcyt.2014.05.005

9. Zhang S, Zhao L, Wang J, Chen N, Yan J, Pan X. HIF-2α and Oct4 have synergistic effects on survival and myocardial repair of very small embryonic-like mesenchymal stem cells in infarcted hearts. Cell Death Dis. 2017; 8: e2548. doi: 10.1038/cddis.2016.480

10. Yin D, Fu C, Sun D. Silence of lncRNA UCA1 represses the growth and tube formation of human microvascular endothelial cells through miR-195. Cell Physiol Biochem. 2018; 49:1499-1511 doi: 10.1159/000493454

11. Bruna A, Greenwood W, Le Quesne J, Teschendorff A, Miranda-Saavedra D, Rueda OM, Sandoval JL, Vidakovic AT, Saadi A, Pharoah P, Stingl J, Caldas C. TGFβ induces the formation of tumour-initiating cells in claudin^low^ breast cancer. Nat Commun. 2012; 3: 1055 doi: 10.1038/ncomms2039

12. Wang JH, Zhao L, Pan X, Chen NN, Chen J, Gong QL, Su F, Yan J, Zhang Y, Zhang SH. Hypoxia-stimulated cardiac fibroblast production of IL-6 promotes myocardial fibrosis via the TGF-β1 signaling pathway. Lab Invest. 2016; 96: 839-852. doi: 10.1038/labinvest.2016.65

13. Zhu P, Wang Y, He L, Huang G, Du Y, Zhang G, Yan X, Xia P, Ye B, Wang S, Hao L, Wu J, Fan Z. ZIC2-dependent OCT4 activation drives self-renewal of human liver cancer stem cells. J Clin Invest. 2015; 125: 3795-808 doi: 10.1172/JCI81979

14. Shen Y, Zhang R, Xu L, Wan Q, Zhu J, Gu J, Huang Z, Ma W, Shen M, Ding F, Sun H. Microarray analysis of gene expression provides new insights into denervation-induced skeletal muscle atrophy. Front Physiol. 2019; 10: 1298. doi: 10.3389/fphys.2019.01298

15. Wang JH, Kumar S, Liu GS. Bulk Gene Expression Deconvolution Reveals Infiltration of M2 Macrophages in Retinal Neovascularization. Invest Ophthalmol Vis Sci. 2021; 62: 22. doi: 10.1167/iovs.62.14.22.

16. Love MI, Huber W, Anders S. Moderated estimation of fold change and dispersion for RNA-seq data with DESeq2. Genome Biol. 2014; 15: 550. doi: 10.1186/s13059-014-0550-8

17. Alencar GF, Owsiany KM, Karnewar S, Sukhavasi K, Mocci G, Nguyen AT, Williams CM, Shamsuzzaman S, Mokry M, Henderson CA, Haskins R, Baylis RA, Finn AV, McNamara CA, Zunder ER, Venkata V, Pasterkamp G, Björkegren J, Bekiranov S, Owens GK. Stem Cell Pluripotency Genes Klf4 and Oct4 Regulate Complex SMC Phenotypic Changes Critical in Late-Stage Atherosclerotic Lesion Pathogenesis. Circulation. 2020; 142: 2045-2059. doi: 10.1161/CIRCULATIONAHA.120.046672

18 Worringer KA, Rand TA, Hayashi Y, Sami S, Takahashi K, Tanabe K, Narita M, Srivastava D, Yamanaka S. The let-7/LIN-41 pathway regulates reprogramming to human induced pluripotent stem cells by controlling expression of prodifferentiation genes. Cell Stem Cell. 2014; 14: 40-52. doi: 10.1016/j.stem.2013.11.001.

19 Loots GG, Ovcharenko I, Pachter L, Dubchak I, Rubin EM. rVista for comparative sequence-based discovery of functional transcription factor binding sites. Genome Res. 2002; 12: 832-939. doi: 10.1101/gr.225502.

20 Gao L, Mei S, Zhang S, Qin Q, Li H, Liao Y, Fan H, Liu Z, Zhu H. Cardio-renal Exosomes in Myocardial Infarction Serum Regulate Proangiogenic Paracrine Signaling in Adipose Mesenchymal Stem Cells. Theranostics. 2020; 10: 1060-1073. doi: 10.7150/thno.37678

21 Zhang S, Zhao L, Shen L, Xu D, Huang B, Wang Q, Lin J, Zou Y, Ge J. Comparison of various niches for endothelial progenitor cell therapy on ischemic myocardial repair: coexistence of host collateralization and Akt-mediated angiogenesis produces a superior microenvironment. Arterioscler Thromb Vasc Biol. 2012; 32: 910-923. doi: 10.1161/ATVBAHA.111.244970

22 Dedkov EI, Zheng W, Christensen LP, Weiss RM, Mahlberg-Gaudin F, Tomanek RJ. Preservation of coronary reserve by ivabradine-induced reduction in heart rate in infarcted rats is associated with decrease in perivascular collagen. Am J Physiol Heart Circ Physiol. 2007; 293: H590-H598. doi: 10.1152/ajpheart.00047.2007

**Supplementary Figure and Table Legends**

**Supplementary Fig. S1. Flow chart of cell preparation, gene transfection, culture, transplantation, echocardiography, and histopathologic evaluation.**

**Supplementary Fig. S2. Characterization of MSCs from myocardial ischemic rats.** **A**, Morphology of cells isolated from the heart and peripheral blood of rats after MI or sham operation. Cells were plastic-adherent and displayed a typical fibroblast-like spindle shape. Representative pictures from each group at passage 1. **B**, Growth of cardiac MSCs and peripheral blood MSCs from rats with myocardial ischemia 30 days after MI or rats after sham operation. Proliferation rate was assessed after 3 passages on the basis of CCK-8 assay by immunofluoroscence in each MSC group. Cardiac MSCs from myocardial ischemic rats displayed the highest growth rate (at 96 hours, *P*<0.001). Results are expressed as the optical density (OD) value for each day per MSC group±SEM. Measures of cell proliferation over time and among groups were analyzed by a 1-way repeated-measures ANOVA and a Tamhane’s T2 multiple comparison test. **C**, Flow cytometry analysis of the immunophenotypic surface profiles for SH2, SH3, CD90, CD147, CD34, CD45, and CD133 of cultured MSCs. Third passage cultured MSCs from the different groups expressed diverse levels for the MSC markers SH2, SH3, CD90, and CD147; but negative for CD34, CD45, and CD133. Data are shown as means ± SEM. A 1-way ANOVA and a Fisher’s multiple comparison test were used for statistical analysis. **D-G** Multilineage differentiation (osteogenic, adipogenic, chondrogenic, and blood vascular) of MSCs from the heart and peripheral blood of MI rats was induced *in vitro,* which was further assessed by specific staining. **C**, Osteogenesis-committed differentiation of MSCs stained by alizarin red (red). **D**, Adipogenesis-committed differentiation of PBMSCs stained by oil red O (red). **E**, Chondrogenesis-committed differentiation of PBMSCs stained by alcian blue (blue). **F**, Staining after normal culture with dye-free solution. **G**, Vascular endothelial cells were examined via immunofluorescence for the expression of ectodermal cell markers, Factor VIII (red) and α-SMA (green). Also shown are DAPI staining (nuclei; blue) and merged images. cMSCs indicate ischemic cardiac mesenchymal stem cells;Isch, ischemic; pbMSCs, peripheral blood mesenchymal stem cells.

**Supplementary Fig. S3. c-Myc and Oct4 contribute differently to the *in vitro* growth of cMSCs. A** and **B**. c-Myc (**A**) and Oct4 (**B**) mRNA expression levels in ischemic cMSCs transfected with a lentivirus encoding overexpressed (*oe*) c-Myc (*oe*c-Myc), Oct4 (*oe*Oct4), or vehicle (served as control, CON) for 48 hours under normoxic or hypoxic conditions. Graphs represent the means ± SEM, and independent samples t test was used (*n* = 5 per group). **C** and **D**, Dynamic analysis of cell proliferation index assessed as the percentage of BrdU-positive cells to total number of cells was determined by FACS in the individual groups at 12-h, 24-h, 36-h, and 48-h after different treatments. All data are the means ± SEM (*n* = 5 per group), and statistical significance relative to normoxia is assessed using two-tailed unpaired Student’s t-test with Welch’s correction. **E** and **F**, A percentage of Ki67 positively stained cells was expressed a proliferative index. **G** and **H**, The growth capacities of cMSCs were also evaluated by CCK-8 assay in optical density (OD) value. Note that all these proliferative indexes of cMSCs showed the greatest levels in the Hypoxia+*oe*Oct4 group, the second highest in the Normoxia+*oe*c-Myc group, and the smallest in the Hypoxia+CON group. Graphs represent the means ± SEM, and independent samples t test was used (*n* = 5 per group). **I** and **J**, Fluorescence microscope images of the cells double stained with DAPI and Ki67, showing the greatest positive staining in the Hypoxia+*oe*Oct4 group, and the lowest in the Hypoxia+CON group.

**Supplementary Fig. S4. Proposed mechanism of Oct4 overexpression induced cytoplasmic translocation of c-Myc**. In cMSCs cotransfected with c-Myc and Oct4, c-Myc was mainly translocated to the cytoplasm, but we did not detect decreased level of total c-Myc. c-Myc shuttles from the nuclei into the cytoplasm after addition of exogenous Oct4. The cooperation between c-Myc and Oct4 activated VEGF signaling, which increased MEndoT and angiogenesis of cMSCs.

**Supplementary Tab.S1. Primers for qRT-PCR of rat tissues.**

**Supplementary Tab. S2. The antibodies forfluorescence activated cell sorting (FACS), western blot (WB), enzyme linked immunosorbent assay (ELISA), and immunofluoroscence (IF)**
