# Supplementary figures and images for: Oct4 cooperates with c-Myc to improve mesenchymal-to-endothelial transition and myocardial repair of cardiac-resident mesenchymal stem cells
Source: Stem Cell Res Ther. 2022 Sep 2;13:445. doi: 10.1186/s13287-022-03120-7 (PMC9438134; doi:10.1186/s13287-022-03120-7)

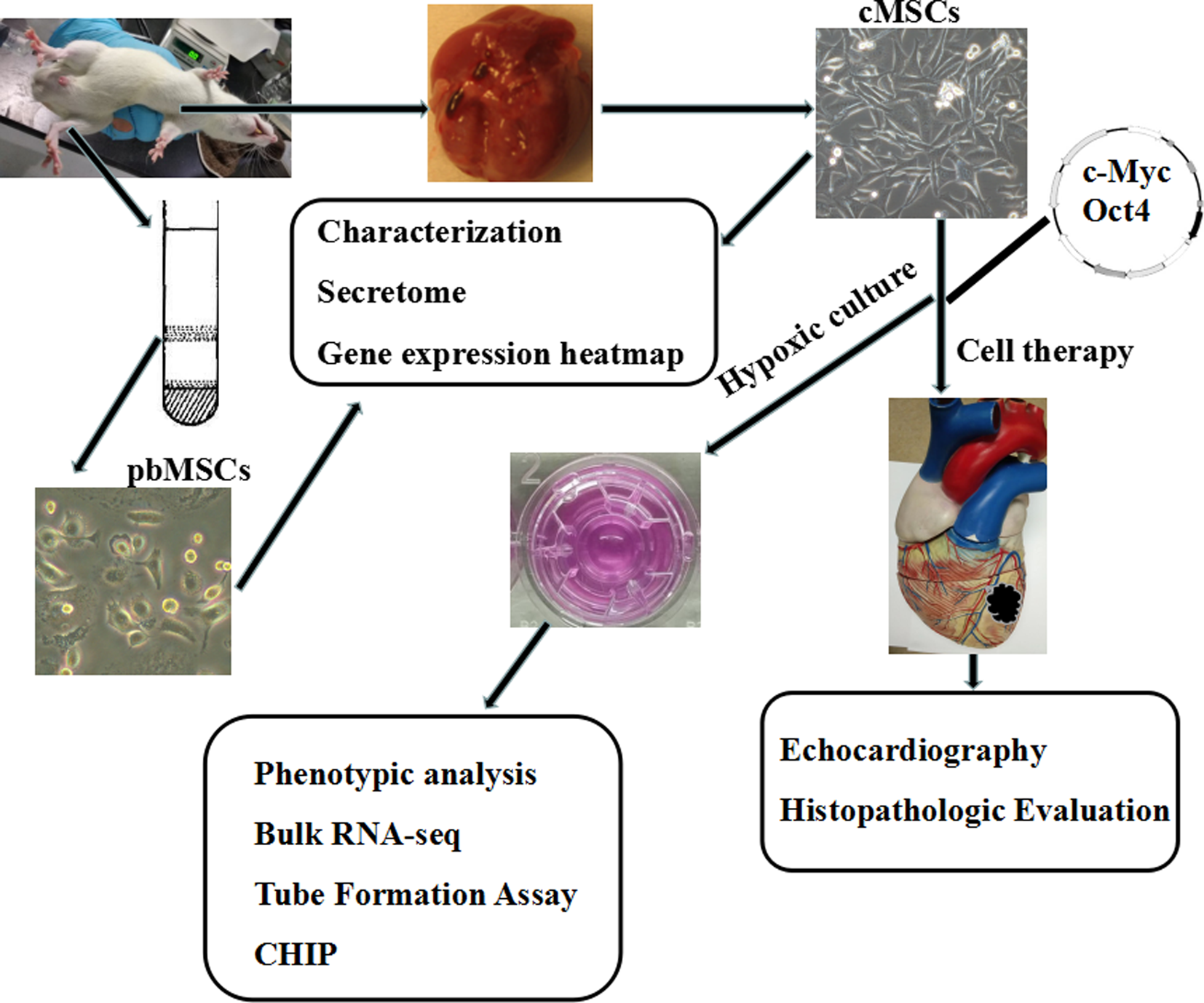

Supplement: Supplementary file 2 — Additional file 2: Fig. S1. Flowchart of cell preparation, gene transfection, culture, transplantation, echocardiography, and histopathologic evaluation. [file 13287_2022_3120_MOESM2_ESM.tif]

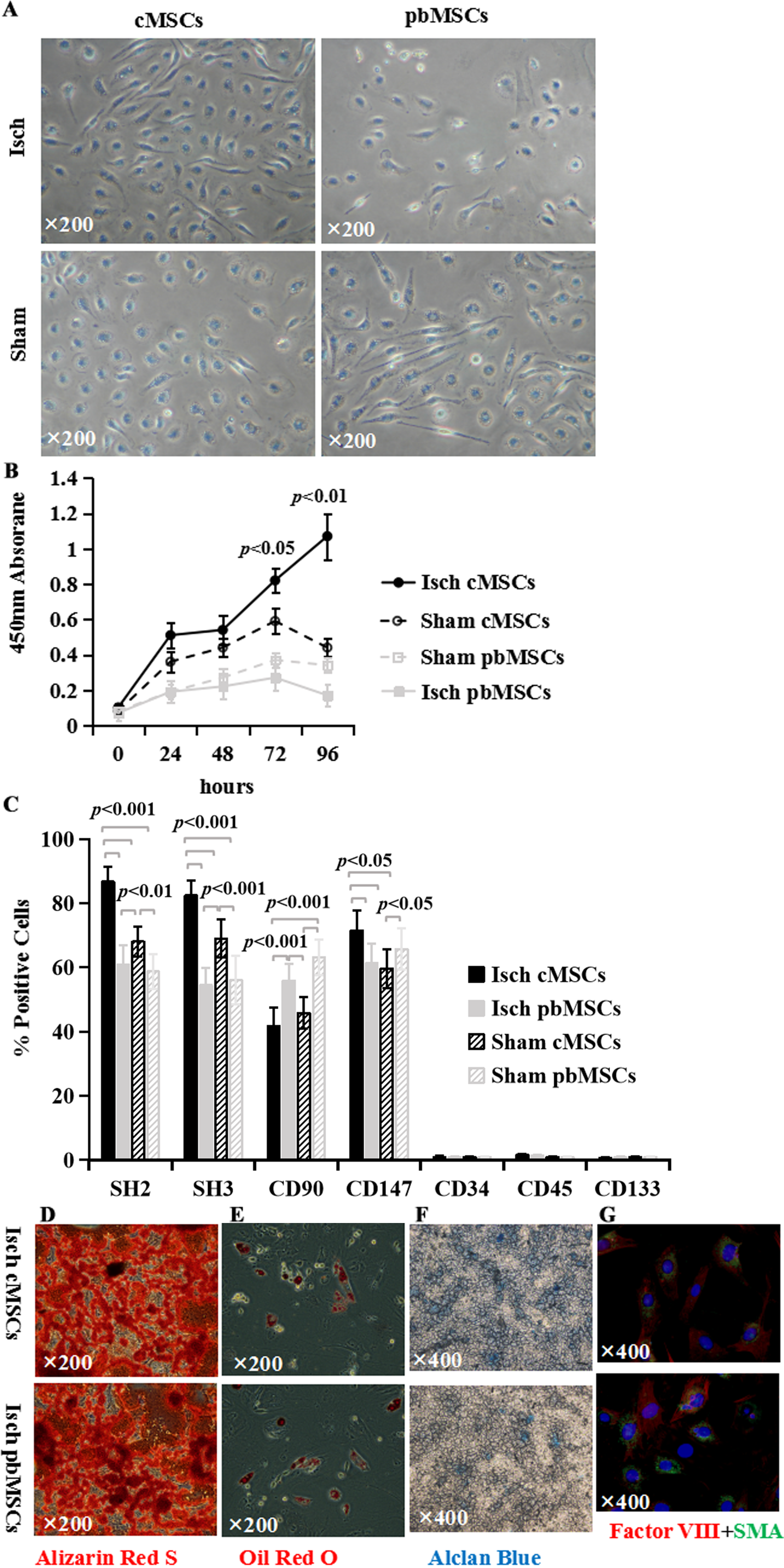

Supplement: Supplementary file 3 — Additional file 3: Fig. S2. Characterization of MSCs from myocardial ischemic rats. [file 13287_2022_3120_MOESM3_ESM.tif]

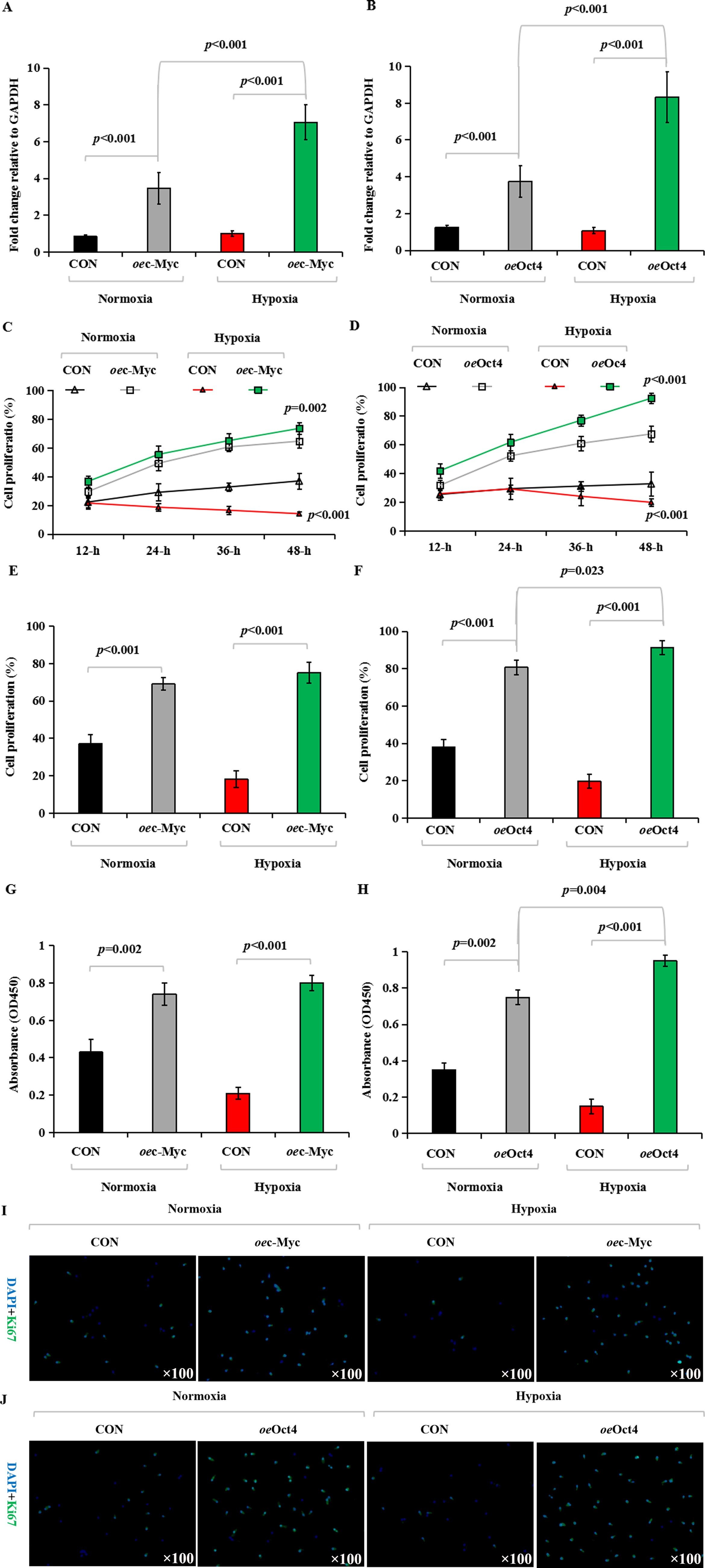

Supplement: Supplementary file 4 — Additional file 4 : Fig. S3. c-Myc and Oct4 contribute differently to the in vitro growth of cMSCs. [file 13287_2022_3120_MOESM4_ESM.tif]

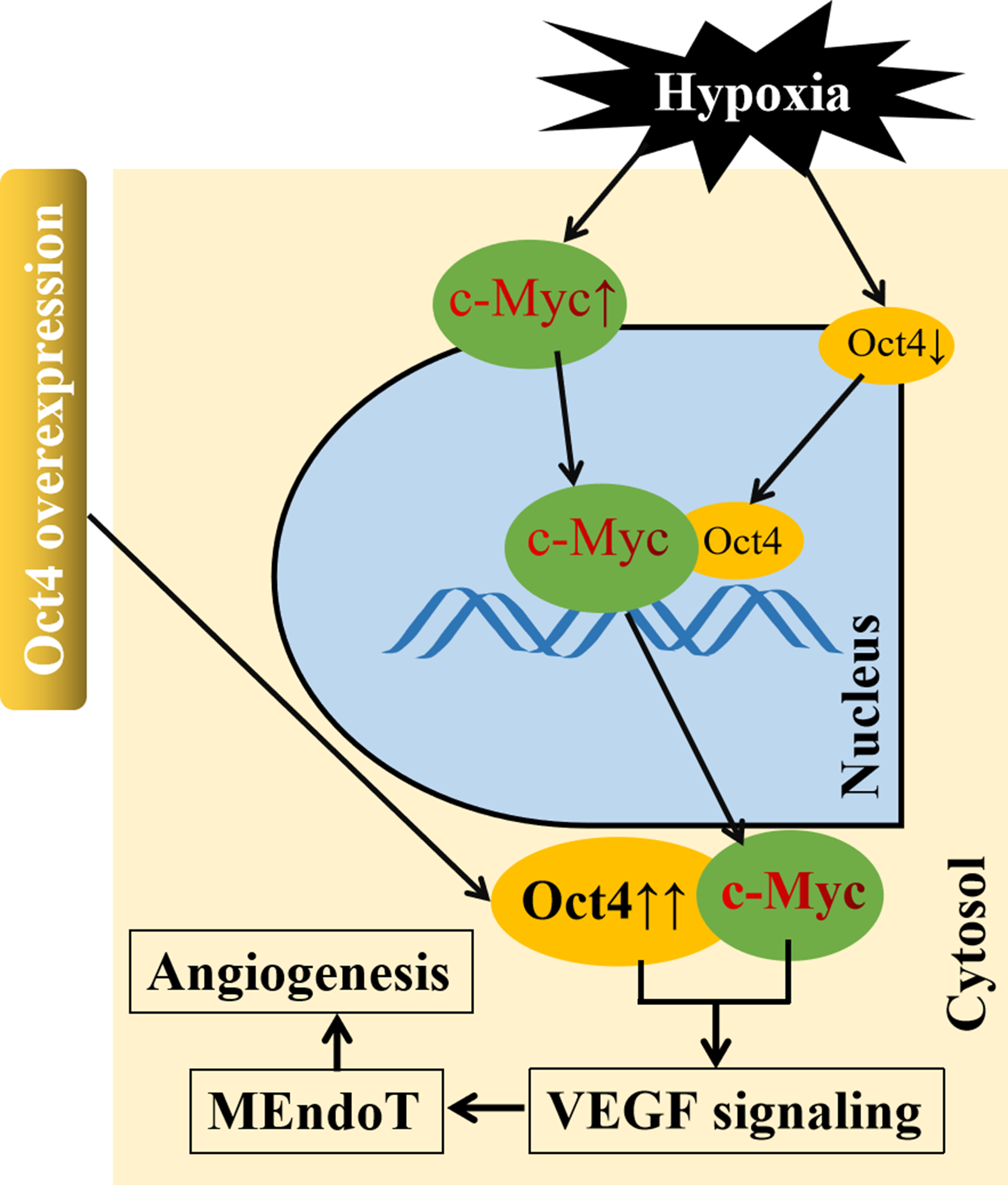

Supplement: Supplementary file 5 — Additional file 5: Fig. S4. Proposed mechanism of Oct4 overexpression induced cytoplasmic translocation of c-Myc. [file 13287_2022_3120_MOESM5_ESM.tif]

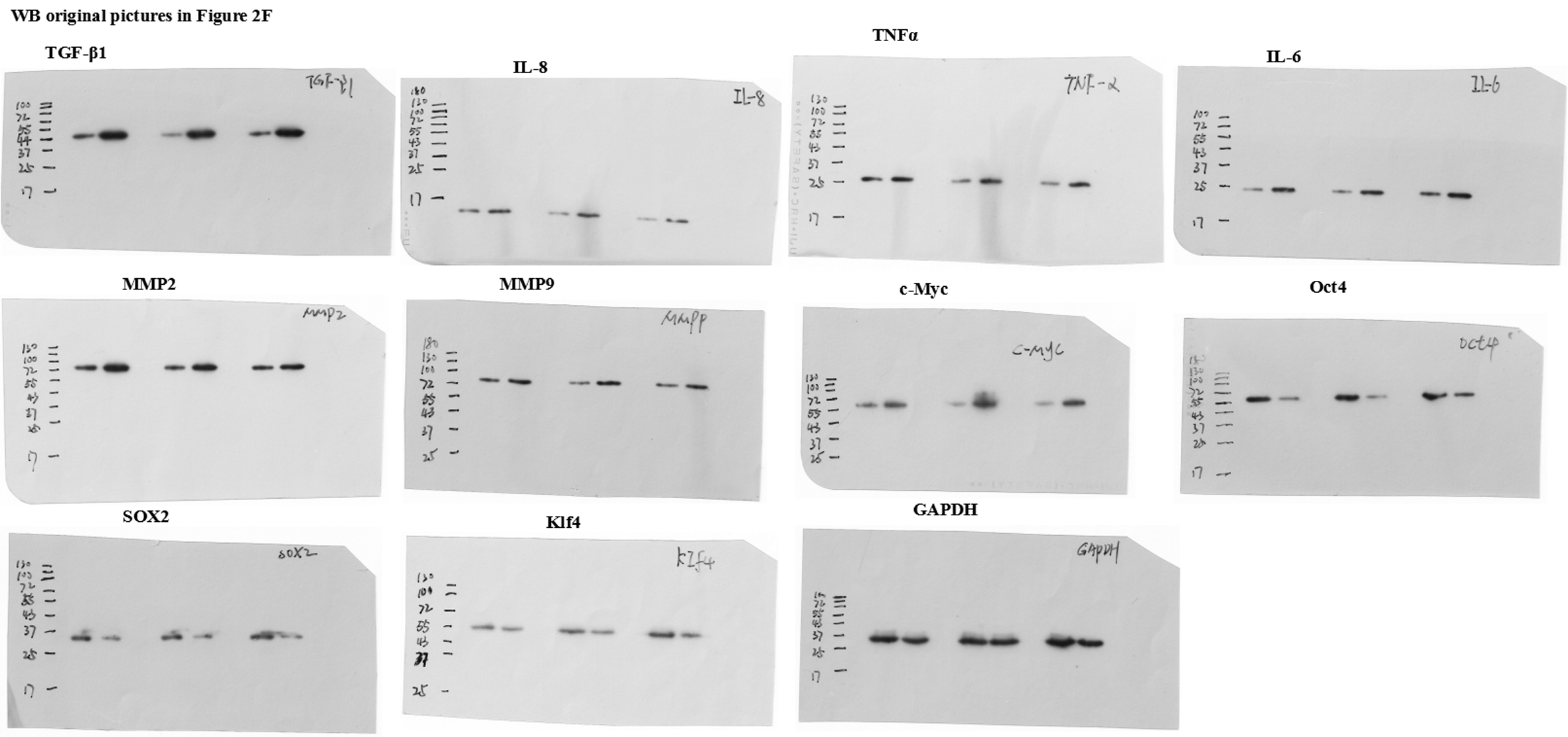

Supplement: Supplementary file 8 — Additional file 8: WB original pictures in Figure 2F. [file 13287_2022_3120_MOESM8_ESM.tif]

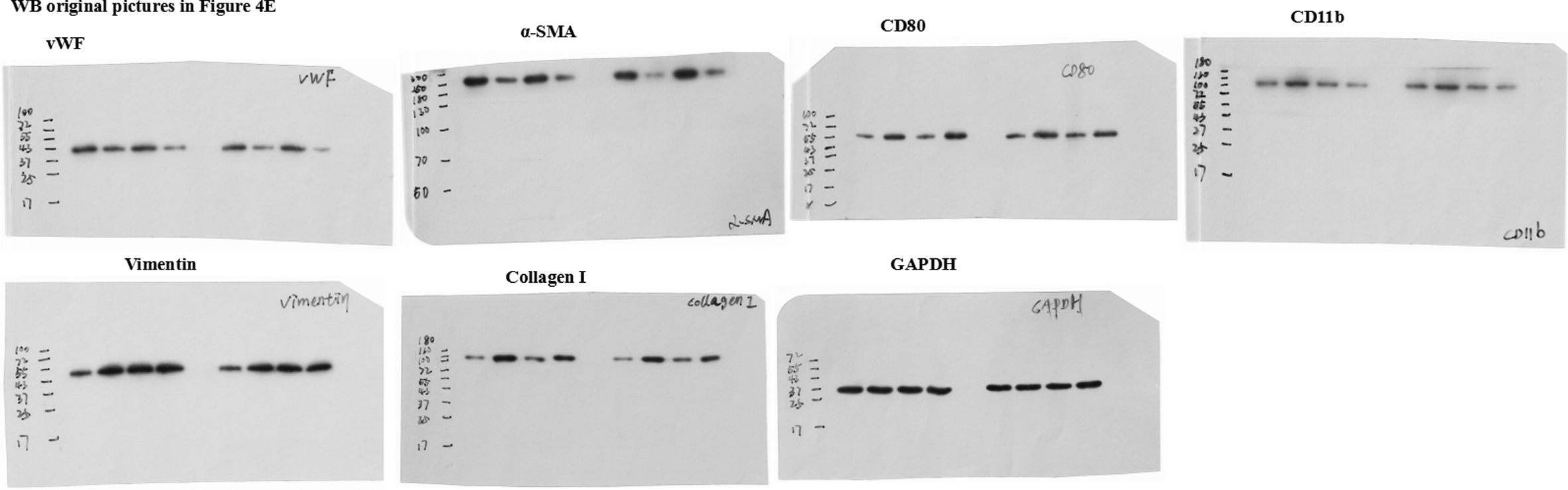

Supplement: Supplementary file 9 — Additional file 9: WB original pictures in Figure 4E. [file 13287_2022_3120_MOESM9_ESM.tif]

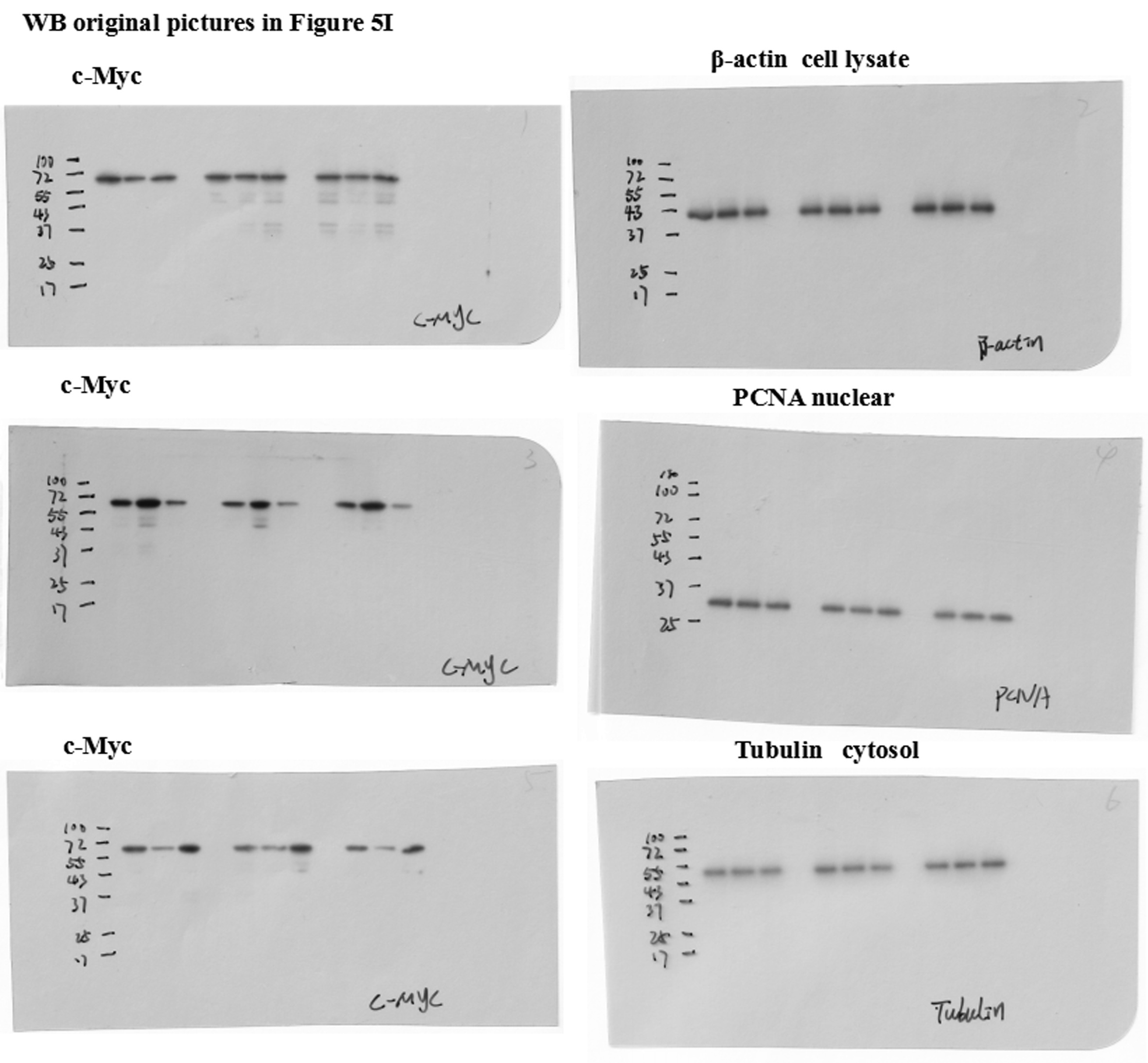

Supplement: Supplementary file 10 — Additional file 10: WB original pictures in Figure 5I. [file 13287_2022_3120_MOESM10_ESM.tif]
