# Supplementary material for: Oct4 cooperates with c-Myc to improve mesenchymal-to-endothelial transition and myocardial repair of cardiac-resident mesenchymal stem cells
Source: Stem Cell Res Ther. 2022 Sep 2;13:445. doi: 10.1186/s13287-022-03120-7 (PMC9438134; doi:10.1186/s13287-022-03120-7)
Supplement: Supplementary file 6 — Additional file 6: Table S1. Primers for qRT-PCR. [file 13287_2022_3120_MOESM6_ESM.doc]

**Table S1 Sequences of primers used for gene amplification.**

| **Genes** | **Forward** | **Reverse** |
| --- | --- | --- |
| Akt | 5′-CACTTTCCCCAGTTCTCCTACT-3′ | 5′- GCCCACAGTAGAAACATCCTC-3′ |
| Ang-1 | 5′-AATGGACTGGGAAGGGAACC-3′ | 5′-GCATCAAACCACCATCCTCCT-3′ |
| Angiogenin | 5′-TCTGGTTTCGACCCCTTCAA -3′ | 5′- CACAGATGGCCTTGATGCTG-3′ |
| bFGF | 5′- AGGCAGGAAGGGAGAAAGTTG-3′ | 5′- GAATCTGTCCCGTTCGGCG-3′ |
| Chd1 | 5′-CGGAGATTAGGCGACCTGAC -3′ | 5′-AAGTCCAGCAAATCAGATGGT -3′ |
| c-Myc | 5′-ATCTCTGGGAGGAATGCTACTA -3′ | 5′-ATCTGCGTGGCTACAGATAAG -3′ |
| c-Myc | 5′-GGGATCCGGAGTCGCAGTAT-3′ | 5′-CTGGAGGCAAAGCCCTTCT-3′ |
| HGF | 5′-AAACTTCTGCCGGTCCTGTT-3′ | 5′-TGGTAAGAGTAGTTTTTGCTGACT-3′ |
| IL-1α | 5′-GCATGGCATGTGCTGAGTCT-3′ | 5′-ATGTCGGGCTGGTTCCACTA-3′ |
| IL-6 | 5′-GCACCTCAGATTGTTGTTG-3′ | 5′-AGTGTCCTAACGCTCATAC-3′ |
| IGF1 | 5′-TGCACCAGAGGTTCTAGGAT -3′ | 5′- TAGCCTGTGGGCTTGTTGAA-3′ |
| IL-8 | 5′-ACCACACTGCGCCAACACAGAAAT-3′ | 5′-TCCAGACAGAGCTCTCTTCCATCAGA -3′ |
| Klf4 | 5′-GGGAAGGGAGAAGACACTGC-3′ | 5′-CCACTTTCCAGGTCTGTGGC-3′ |
| MAPK | 5′- CCTCCTCCCTTGCATTCAGAAC-3′ | 5′-AGAGCCAGGGCTATCTGCTTTG -3′ |
| MMP2 | 5′-TTGACGGTAAGGACGGACTC-3′ | 5′-GGCGTTCCCATACTTCACAC-3′ |
| MMP9 | 5′-AAGGGCGTCGTGGTTCCAACTC-3′ | 5′-AGCATTGCCGTCCTGGGTGTAG-3′ |
| Oct4 | 5′-CCTTGTCCCACTTGCTGGTT-3′ | 5′-AGCAAAGACGGGGCAAGAAA-3′ |
| Sox2 | 5′-AGTGGTACGTTAGGCGCTTC -3′ | 5′- ATCGCCCGGAGTCTAGTTCT-3′ |
| TGF-β1 | 5′-GACTACTACGCCAAGGAGGTC-3′ | 5′-GAGAGCAACACGGGTTCAG-3′ |
| Tie2 | 5′-AGATGTGTGACCGCTTCCAA -3′ | 5′-ATCCTTGGCCTGCCTTGCTT -3′ |
| TNFα | 5′- ATGGAATCCGGCTCAACAGG-3′ | 5′- CACCACACTCCTTTGCGTTC-3′ |
| VEGF | 5′-CCGACAGGGAAGACAATGGGA-3′ | 5′-GGGATGGGTTTGTCGTGTTTCT-3′ |
| VEGFR | 5′-TCTTGTCTCTGGCGTGTTCC -3′ | 5′-GCCTTCGAAACCCTTGACCT -3′ |
| vWF | 5′-CTACGTAGAGCGTGAGGCTG -3′ | 5′-TCTGAGGTCAAGGTCCCCTC -3′ |
| GAPDH | 5′- GCATCTTCTTGTGCAGTGCC-3′ | 5′- TACGGCCAAATCCGTTCACA-3′ |
