# Supplementary material for: Oct4 cooperates with c-Myc to improve mesenchymal-to-endothelial transition and myocardial repair of cardiac-resident mesenchymal stem cells
Source: Stem Cell Res Ther. 2022 Sep 2;13:445. doi: 10.1186/s13287-022-03120-7 (PMC9438134; doi:10.1186/s13287-022-03120-7)
Supplement: Supplementary file 7 — Additional file 7: Table S2. The antibodies for western blotting, immunofluorescence, and immunohistochemistry, respectively. [file 13287_2022_3120_MOESM7_ESM.doc]

**Table S 2 The antibodies for For Complexometry  (FCM), western blot (WB), enzyme linked immunosorbent assay (ELISA), and immunofluoroscence (IF)**

| Name | Description | Company | Catalogue number | Applications |
| --- | --- | --- | --- | --- |
| α-SMA | Alpha-smooth muscle actin | Millipore | MAB1522 | WB |
| Ang1 | Angiopoietin 1 | Biorbyt  [RayBiotech](https://www.biocompare.com/100815-Raybiotech-Inc/) | orb10091 ELR-Angiopoietin1-1 | WB, IF, ELISA |
| Bax | BCL2-Associated X | [GeneTex](https://www.biocompare.com/100423-GeneTex/) | GTX109683 | WB, IF, ELISA |
| β-Actin | Beta Actin | [GeneTex](https://www.biocompare.com/100423-GeneTex/) | GTX33610 | WB, IF, ELISA |
| Bcl2 | B-cell lymphoma-2 | [Abcam](http://www.biocompare.com/9776-Antibodies/2275835-Bcl2-antibody/?firofb=true&pda=9776|2275835_0_0|1014,3687,3681,887|6|Bcl2) | ab692 | WB, IF, ELISA |
| BCLF1 | Bcl-2-associated transcription factor 1 | [MyBioSource](https://www.biocompare.com/104355-MyBioSource-com/) | MBS851985 | WB, ELISA |
| BrdU |  | [Enzo Life Sciences](https://www.biocompare.com/100040-Enzo-Life-Sciences-Inc/) | ADI-MSA-200-E | FCM, IF |
| bFGF | Basic fibroblast growth factor | [MyBioSource](https://www.biocompare.com/104355-MyBioSource-com/)  [RayBiotech](https://www.biocompare.com/100815-Raybiotech-Inc/) | MBS551041  ELR-bFGF-1 | WB, IF, ELISA |
| Caspase-3 |  | Abcam | ab63399 |  |
| CD31 | Platelet and endothelial cell adhesion molecule 1 (PECAM1) | [ABclonal Technology](https://www.biocompare.com/106407-ABclonal/) | A2104 | WB, IF |
| CD71 | Cluster of differentiation 71 | [MyBioSource](https://www.biocompare.com/104355-MyBioSource-com/) | MBS212212 | FCM, WB |
| CD34 | Cluster of differentiation 34 | [MyBioSource](https://www.biocompare.com/104355-MyBioSource-com/) | MBS520138 | FCM, IF |
| CD45 | Cluster of differentiation 45 | [MyBioSource](https://www.biocompare.com/104355-MyBioSource-com/) | MBS438123 | FCM |
| CD90 | Cluster of differentiation 90 | [MyBioSource](https://www.biocompare.com/104355-MyBioSource-com/) | MBS213035 | FCM, WB |
| CD80 | Cluster of differentiation 80 | [ABclonal Technology](https://www.biocompare.com/106407-ABclonal/) | A16039 | WB, IF |
| CD147 | Cluster of differentiation 147 | [MyBioSource](https://www.biocompare.com/104355-MyBioSource-com/) | MBS249247 | FCM, WB |
| CD133 | Cluster of differentiation 133 | [MyBioSource](https://www.biocompare.com/104355-MyBioSource-com/) | MBS462020 | FCM, WB |
| CD11b | Cluster of differentiation 11b | LifeSpan BioSciences  [MyBioSource.com](https://www.biocompare.com/104355-MyBioSource-com/) | LS-C45059  MBS176783 | IF  WB |
| c-Fos |  | [MyBioSource](https://www.biocompare.com/104355-MyBioSource-com/) | MBS8510383 | WB,IF |
| c-Myc |  | NSJ Bioreagents | RQ4860 | WB, IF |
| Collagen I |  | Abcam | ab84956 | WB, IF |
| Factor VIII | Von willebrand factor (vWF) | [Biorbyt](http://www.biocompare.com/9776-Antibodies/2312352-Factor-VIII/?soids=3686,257&ppim=2312352_1_1&ncatid=9776&dfp=true#%23) | orb10633 | WB, IF |
| FoxC1 | Forkhead box C1 | [Aviva](https://www.biocompare.com/9776-Antibodies/10647563-FOXC1-Antibody-middle-region-OASG02780/?pda=9776|10647563_0_1|1014,3686,887|4|FOXC1&dfp=true) | OASG02780 | WB, IF |
| FoxO1 | Forkhead box O1 | [MyBioSource](https://www.biocompare.com/104355-MyBioSource-com/) | MBS8222615 | WB, IF |
| GAPDH | Glyceraldehyde-3- phosphate dehydrogenase | [Novus Biologicals](http://www.biocompare.com/9776-Antibodies/119146-GAPDH-Antibody/?soids=1014,257&ppim=119146_1_1&ncatid=9776&dfp=true#%23) | NB300-221 | WB |
| HIF-1α | Hypoxia inducibel factor 1 alpha | MyBioSource | MBS477549 | WB |
| HGF | Hepatocyte growth factor | [RayBiotech](https://www.biocompare.com/100815-Raybiotech-Inc/)  [MyBioSource](https://www.biocompare.com/104355-MyBioSource-com/) | ELR-HGF-1  MBS177652 | ELISA, WB |
| Ki-67 |  | Abcam | ab15580 | IF |
| Klf4 | Kruppel-like factor 4 | Boster | BA3453 | WB,IF |
| IL-1α | Interleukin-1alpha | [Santa Cruz Biotechnology](https://www.biocompare.com/100404-Santa-Cruz-Biotechnology-Inc/) | sc-12741 | WB,IF, FCM, ELISA |
| IL-4 | Interleukin-4 | [RayBiotech](https://www.biocompare.com/100815-Raybiotech-Inc/) | ELR-IL4-1 | ELISA |
| IL-6 | Interleukin-6 | Abcam  [RayBiotech](https://www.biocompare.com/100815-Raybiotech-Inc/) | ab6672  ELR-IL6-1 | WB, IF  ELISA |
| IL-10 | Interleukin-10 | [RayBiotech](https://www.biocompare.com/100815-Raybiotech-Inc/) | ELR-IL10-1 | ELISA |
| Laminin |  | [OriGene Technologies](https://www.biocompare.com/100314-OriGene-Technologies/) | BM6046P | IF, FCM |
| MMP2 | Matrix metalloproteinase2 | Abcam | ab2462 | WB |
| MMP9 | Matrix metalloproteinase9 | Abcam | ab38898 | WB |
| Nanog |  | Novus Biologicals | NBP1-77109 | WB, IF |
| Oct4 | Octamer‑binding protein 4 | [LifeSpan BioSciences](https://www.biocompare.com/100534-LifeSpan-BioSciences/) | LS-C118538 | IF, WB, ELISA |
| PCNA |  |  |  |  |
| SH2 | Src homology domain 2 | [LifeSpan BioSciences](https://www.biocompare.com/100534-LifeSpan-BioSciences/) | LS-B7922 | FCM, WB |
| SH3 | Src homology domain 3 | Antibodies-  online | ABIN5542542 | FCM, IF, WB, ELISA |
| SMA | alpha Smooth Muscle Actin | [MyBioSource.com](https://www.biocompare.com/104355-MyBioSource-com/) | MBS8511177 | WB, IF |
| SOX2 | SRY-box 2 | [MyBioSource.com](https://www.biocompare.com/104355-MyBioSource-com/) | MBS9408981 | WB, IF |
| TGF-β1 | Transforming srowth factor beta-1 | [GeneTex](https://www.biocompare.com/100423-GeneTex/) | GTX45121 | IF, WB |
| Tie2 |  | [Proteintech Group Inc](https://www.biocompare.com/102174-Proteintech-Group-Inc/) | 19157-1-AP | IF, WB, ELISA |
| TNFα | Tumor necrosis factor alpha | [GeneTex](https://www.biocompare.com/100423-GeneTex/) | GTX110520 | WB, IF |
| Vimentin |  | [GeneTex](https://www.biocompare.com/100423-GeneTex/) | GTX100619 | IF, WB, ELISA |

| TGFβ1 | Transforming growth factor beta1 | [Biorbyt](https://www.biocompare.com/104369-Biorbyt/) | orb225553 | IF, WB, ELISA |
| --- | --- | --- | --- | --- |
| VEGF | Vascular endothelial growth factor | Bioss Inc.  [Biorbyt](https://www.biocompare.com/104369-Biorbyt/) | bs-0279R  orb566706 | WB, IF, ELISA |
| VEGFR2 | [Vascular endothelial growth factor receptor 2](https://www.biocompare.com/9776-Antibodies/7559527-VEGFR2-Antibody/?pda=9776|7559527_0_0|269752,1014,2,3687,3681,887|10|VEGFR2) | [Novus Biologicals](https://www.biocompare.com/100434-Novus-Biologicals/) | NBP2-89200-100ul | ELISA |
| Vimentin |  | [MyBioSource](https://www.biocompare.com/104355-MyBioSource-com/) | MBS500042 | WB, IF |
